# Cost-effectiveness of weight-management pharmacotherapies in Canada: a societal perspective

**DOI:** 10.1038/s41366-024-01467-w

**Published:** 2024-01-31

**Authors:** Anamaria-Vera Olivieri, Sergey Muratov, Sara Larsen, Maria Luckevich, Katalina Chan, Mark Lamotte, David C. W. Lau

**Affiliations:** 1 QVIA, Basel, Switzerland; 2 QVIA Mississauga, Mississauga, ON Canada; 3 Novo Nordisk A/S, Søborg, Bagsværd, Denmark; 4 Novo Nordisk Canada Inc, Mississauga, ON Canada; 5 QVIA, Zaventem, Zaventem, Belgium; 6 Department of Medicine, University of Calgary Cumming School of Medicine, Calgary, AB Canada

**Keywords:** Health care, Risk factors

## Abstract

**Objectives:**

This study aimed to assess the cost-effectiveness of weight-management pharmacotherapies approved by Canada Health, i.e., orlistat, naltrexone 32 mg/bupropion 360 mg (NB-32), liraglutide 3.0 mg and semaglutide 2.4 mg as compared to the current standard of care (SoC).

**Methods:**

Analyses were conducted using a cohort with a mean starting age 50 years, body mass index (BMI) 37.5 kg/m^2^, and 27.6% having type 2 diabetes. Using treatment-specific changes in surrogate endpoints from the STEP trials (BMI, glycemic, blood pressure, lipids), besides a network meta-analysis, the occurrence of weight-related complications, costs, and quality-adjusted life-years (QALYs) were projected over lifetime.

**Results:**

From a societal perspective, at a willingness-to-pay (WTP) threshold of CAD 50 000 per QALY, semaglutide 2.4 mg was the most cost-effective treatment, at an incremental cost-utility ratio (ICUR) of CAD 31 243 and CAD 29 014 per QALY gained versus the next best alternative, i.e., orlistat, and SoC, respectively. Semaglutide 2.4 mg extendedly dominated other pharmacotherapies such as NB-32 or liraglutide 3.0 mg and remained cost-effective both under a public and private payer perspective. Results were robust to sensitivity analyses varying post-treatment catch-up rates, longer treatment durations and using real-world cohort characteristics. Semaglutide 2.4 mg was the preferred intervention, with a likelihood of 70% at a WTP threshold of CAD 50 000 per QALY gained. However, when the modeled benefits of weight-loss on cancer, mortality, cardiovascular disease (CVD) or osteoarthritis surgeries were removed simultaneously, orlistat emerged as the best value for money compared with SoC, with an ICUR of CAD 35 723 per QALY gained.

**Conclusion:**

Semaglutide 2.4 mg was the most cost-effective treatment alternative compared with D&E or orlistat alone, and extendedly dominated other pharmacotherapies such as NB-32 or liraglutide 3.0 mg. Results were sensitive to the inclusion of the combined benefits of mortality, cancer, CVD, and knee osteoarthritis.

## Introduction

A body mass index (BMI) of 25–29.9 kg/m² or ≥30 kg/m² and 23–24.9 kg/m² or ≥25 kg/m² in White, Hispanic, Black individuals and Asian populations, respectively is classified as overweight or obesity [1]. In Canada, 7.3 million adults (26.8%) are categorized as having obesity, and a further 9.9 million adults (36.3%) as living with overweight [2]. Overweight and obesity pose a huge economic burden. The World Obesity Report estimated that, in 2019, the total economic burden associated with overweight, and obesity was US$ 40.3 billion in Canada. This accounted for US$ 1 078 per capita and 2.3% of the gross domestic product (GDP). Direct health care costs were estimated at US$ 14.8 billion, while indirect costs (including premature mortality costs, absenteeism costs, and presenteeism costs) represented 63.4% (i.e., US$ 25.7 billion) of the total costs. By 2060, this economic impact is predicted to increase fourfold, to US$ 162.35 billion (total direct costs: US$ 41.19 billion and total indirect costs: US$ 121.15 billion), which will be equivalent to US$ 3398 per capita and 3.7% of the GDP [3].

Obesity is a complex, chronic disease mediated by genetic, physiological, environmental, and psychological factors [4–7]. Adiposity markers such as body weight, BMI, waist circumference, etc. have been shown in population-wide observational studies and, more recently, in large-scale genetic analyses to be causally related to higher rates of type 2 diabetes (T2D), coronary heart disease (CHD), ischemic stroke, sleep apnea, osteoarthritis, and certain cancers [2, 8, 9]. However, a limited number of studies have investigated whether weight-loss can reduce the incidence/prevalence of such complications. Reduction in overall mortality as well as lower incidence of myocardial infarction (MI), stroke, cancer in females; and diabetes remission were observed for 20 years following bariatric surgery (BaS) in the Swedish Obese Subjects trial [10]. Furthermore, a population-based study of the United Kingdom (UK) Clinical Practice Research Datalink (CPRD) GOLD showed that a median weight-loss of 13% during 1–4 years was associated with a reduction in T2D, hypertension, dyslipidemia, and kidney disease but not CHD or overall mortality to levels below those measured in individuals who had maintained a correspondingly lower BMI at 10.5 years from the study index date [11]. Delays in T2D onset and reductions in sleep apnea prevalence [12] were also shown in weight-loss pharmacological studies. In SCALE study, liraglutide 3.0 mg, reduced baseline weight by 4.3% (95% CI [confidence interval] -4.9 to -3.7), compared to diet and exercise (D&E). After 3 years of weight-management treatment, sixty-six percent of patients on liraglutide 3.0 mg experienced a glycemic-normalization effect, compared with 36% in the placebo group (odds ratio [OR] 3.6, 95% CI 3.0–4.4) [13]. This resulted in a reduction in T2D onset by 79% (hazard ratio [HR] 0.21, 95% CI 0.13–0.34) in individuals with overweight, obesity, and prediabetes. A delay in the onset or reversal of T2D was also demonstrated with intensive lifestyle modification and diet [14–16], orlistat, or phentermine/topiramate [17, 18]. To date, no pharmacological interventional studies have demonstrated a decrease in the incidence of major, acute, cardiovascular (CV) events, cancer, mortality, or surgeries due to osteoarthritis in obesity.

There are four pharmacological agents approved for chronic weight-management by Health Canada, all of which are recommended for use in addition to medical nutrition and exercise therapy, i.e., D&E: semaglutide 2.4 mg (approved in November 2021) [19]; liraglutide 3.0 mg (February 2015); naltrexone 32 mg/bupropion 360 mg (NB-32, March 2018) in a combination; and orlistat (in 1999) [20–22]. Subcutaneous semaglutide 2.4 mg injection (Wegovy^®^) is a GLP-1 receptor agonist (GLP1-RA) approved for the treatment of obesity and overweight with ≥1 weight-related disease in Canada [19]. Semaglutide Effects on Heart Disease and Stroke in Patients With Overweight or Obesity (SELECT) is the first study to investigate the effects of semaglutide 2.4 mg on CV and mortality endpoints in this population. The trial’s completion is expected in September 2023 [23, 24]. A CV-protective benefit was demonstrated with semaglutide (0.5 mg or 1.0 mg) in a high-risk population with T2D and a baseline mean BMI of 32.8 kg/m^2^ [25]. Other CV outcome trials in overweight/obesity pharmacotherapy are ongoing, and the results are expected in 2024 [26]. NB-32 can lead to an increase in blood pressure (BP) and heart rate, making it challenging to prescribe to patients with significant CV disease. The failure of two CV outcome trials (CVOT) to assess CV safety of NB-32 represents a considerable setback [27]. On the contrary, orlistat and liraglutide 3.0 mg are considered appropriate for people with CV disease. A long-term CVOT (LEADER trial) of patients with T2D and at a high-risk of having CV-events showed superiority of liraglutide 1.8 mg over the placebo in the primary outcome (e.g., time of CV death, non-fatal MI, and non-fatal stroke) [28]. BaS is reserved for individuals with BMI ≥ 40 kg/m^2^ or BMI ≥ 35 kg/m^2^ and weight-related diseases, but historically, only people with much higher BMIs have had access to this procedure in Canada [29, 30].

This study aimed to assess the cost-effectiveness of weight-management pharmacotherapies approved by Canada Health and recommended by the Canadian Adult Obesity Clinical Practice Guidelines, i.e., orlistat, NB-32, liraglutide 3.0 mg and semaglutide 2.4 mg compared to the current standard of care (SoC) with D&E [31]. BaS was considered a next-line therapy in a scenario analysis. Treatment changes in surrogate clinical endpoints, known to represent risk factors for weight-related diseases, were modeled using a published cost-effectiveness model, namely the Core Obesity Model (COM) to result in a reduction or delay of weight-related diseases, i.e., hard endpoints. Therefore, the current analyses may represent an early evaluation of cost-effectiveness for orlistat, NB-32, liraglutide 3.0 mg and semaglutide 2.4 mg, as the effects of these treatments on certain hard endpoints herein modeled, like the prevention of cancers, cardiovascular events, knee replacement surgeries and mortality have not yet been proven in randomized controlled trials of the studied population. These analyses should be updated along with the emerging evidence on the effects of these treatments on hard clinical endpoints.

## Methods

### Perspective

In the Canadian health care system, multiple stakeholders—including public payers, private insurers, employers, and patients—bear the costs of health care [32]. Canada is one of the few Organization for Economic Co-operation and Development (OECD) countries that do not have a universal public drug benefit coverage [33]. A considerable portion of the outpatient drug costs for disease management are covered by private payers or out of pocket. Services, such as home care, are heavily subsidized for qualifying patients but still involve co-payments to an extent [34]. Hospital care and physician services are fully funded publicly. Moreover, given the mean age of patients with obesity qualifying for treatment is below the retirement age in Canada (i.e., 65 years), costs of productivity losses are anticipated. Therefore, it was deemed relevant to conduct the analyses from the societal perspective in the base-case. In addition, cost-effectiveness results are also presented disaggregated for public and private payers (the latter including employer-based insurances and/or patients’ co-payments) in 2021 Canadian dollars (CAD), discounted at 1.5%. A willingness-to-pay (WTP) threshold of CAD 50 000 per QALY gained was considered, which is an assumption commonly used in Canada for standard health technology assessment (HTA) [35].

### Target population

The target population of the current analysis represents people eligible for treatment with semaglutide 2.4 mg in Canada, namely adults with BMI ≥ 30 kg/m^2^ or 27–30 kg/m^2^ and ≥1 weight-related condition, including T2D. Since the cost-effectiveness is evaluated in a single, closed-cohort, the cohort’s mean baseline characteristics (age, gender, BMI, systolic blood pressure [SBP], lipids, and glycated hemoglobin [HbA_1c_] [for those with baseline T2D]) were derived by weighting the mean baseline characteristics of patients enrolled in two randomized controlled trials (RCTs), namely the Semaglutide Treatment Effect in People Suffering From Overweight or Obesity (STEP 1) and People With Type 2 Diabetes Suffering From Overweight or Obesity (STEP 2) [36, 37]. The weighting of the two studies’ baseline characteristics was done to reflect the real-life distribution of glycemic status and allowed combining the two studies’ results. Thus, 26.0%, 46.4%, and 27.6% of the cohort had normal glucose tolerance (NGT), prediabetes, and T2D at baseline, respectively [2, 38–41]. The resulting baseline characteristics of the combined cohort were a mean starting age of 50 years, mean BMI of 37.5 kg/m^2^, and 67% female (Supplementary Table 1). The inclusion/exclusion criteria of the 2 RCTs are reported elsewhere [36, 37]. For the proportion of the cohort with T2D at baseline, the mean baseline duration of diabetes was 8 years, and the mean HbA_1c_ was 8.1% [37] (Supplementary Table 1).

### Clinical efficacy and safety

The change in BMI, SBP, glycemic status (Supplementary Table 2), total cholesterol, and high-density lipoprotein cholesterol (Supplementary Table 3) were taken from STEP 1 and STEP 2, as observed at Weeks 28 and 68 at baseline in early responders (ERs) to semaglutide 2.4 mg (via a post-hoc analysis in the proportion of patients achieving ≥5% weight-loss vs. baseline), and from all patients in the D&E arm. Non-responders to pharmacotherapy were assumed to discontinue treatment but remain on D&E lifetime, thus were attributed the efficacy of D&E in the STEP trials. This approach is supported by Canadian clinical practice guidelines and reflects the SoC, whereby D&E therapy is considered foundational [42] while pharmacotherapy is only continued in people with clinically meaningful weight-loss after 3 to 6 months on therapeutic dose [31, 43]. In cases in which patients experience additional therapeutic benefits, such as glycemic control, pharmacotherapy may not be stopped on the sole basis of not having met a weight loss threshold, and, for this reason, scenarios were also conducted without the stopping rule, reflecting a more conservative point of reference.

The treatment policy estimand (i.e., population-level treatment effect regardless of treatment adherence and/or initiation of other anti-obesity therapies) was used in all base-case analyses. A scenario was conducted whereby a proportion of the cohort was assumed to discontinue treatment each cycle, in addition to the non-responder discontinuation, and revert to the efficacy of the D&E arm post discontinuation. The trial product estimand efficacy (i.e., efficacy as if all patients adhered to the treatment regimen) was applied to the proportion of cohort remaining on treatment (Supplementary Tables 4, 5). The per cycle discontinuation rates were sourced from the STEP 1 and STEP 2 (Supplementary Table 5). Results from the longer, 104-week RCT, STEP 5 [44], provided information with regards to the maintenance of weight-loss, SBP, and prediabetes reversal for treatment durations beyond those observed in STEP 1 and STEP 2 (Supplementary Table 6). Next-line BaS was applied when the average BMI reached the eligibility threshold of 35 kg/m^2^.

The relative efficacy of other pharmacotherapies was informed via a published network meta-analysis (NMA), based on a systematic literature review conducted in September 2020 [45]. Comprehensive networks were generated by considering outcome data reported at 52–68 weeks (overall 52-week maintenance treatment across all studies after variable titration periods across trials) [45]. Bayesian framework and Markov chain Monte Carlo were used and the results of fixed effects models by subpopulations with NGT, prediabetes, and T2D were used in the cost-effectiveness analysis (CEA). In general, the network of evidence was considered sufficiently homogeneous for the analysis to be conducted, albeit some heterogeneity between studies was noted (on baseline age, sex, and BMI), as well as a risk of bias, due to differences in handling missing data. An NMA published by Shi et al. in 2022 showed consistent results [46]. The head-to-head RCT STEP 8 comparing semaglutide 2.4 mg with liraglutide 3.0 mg was used in a scenario analysis, given that this study was published after the conduct of the NMA. Also, the trial was conducted in a subset of the target population for semaglutide 2.4 mg in Canada without T2D with a mean starting BMI of 37.1 kg/m^2^ (Supplementary Table 1).

The relative efficacy of orlistat, NB-32 and liraglutide 3.0 mg was anchored to the efficacy observed in ERs to semaglutide 2.4 mg. This is in alignment with liraglutide 3.0 mg and NB-32 product labels, whereby treatment is stopped if a ≥ 5% weight-loss is not achieved within 12 weeks on maintenance dose, and with Canadian clinical practice guidelines on pharmacotherapeutic weight-management [31, 43].

Severe gastrointestinal adverse events (AEs), typically associated with weight-management treatments (e.g., nausea, vomiting, diarrhea, and constipation), were included in base-case analyses. Severe and non-severe hypoglycemic events were also included for GLP-1 RA. AEs were applied in the model for as long as the cohort remained on treatment. Serious AEs outside the gastrointestinal tract (such as gallbladder disorders, hepatic disorders, and pancreatitis) were not included in the analyses, given their low frequency [36], expected low impact on costs and utilities in an average cohort, and uncertainty/risk of bias reported in the NMA [45] (Supplementary Table 7).

### Modeling approach

The CEA was conducted using Version 18 of the COM, a validated Markov state-transition model (Supplementary Fig. 1) [47, 48]. The model, designed to evaluate the costs and health outcomes of developing known weight-related complications as a function of risk factors such as BMI, lipids, SBP, and glycemic levels/status, has been fully described elsewhere [47, 48]. Briefly, the occurrence of chronic and/or recurring weight-related complications such as T2D, sleep apnea, acute coronary syndrome (ACS, including MI and unstable angina [UA]), stroke, transient ischemic attacks, post-menopausal endometrial and breast cancers, colon cancer, and knee osteoarthritis surgery were predicted over a time horizon of 40 years (Supplementary Fig. 1). Treatment effects on comorbidities were modeled via changes in surrogate endpoints known to increase their risk (e.g., BMI, SBP, glycemia, and lipids). The latter were evaluated in clinical trials, while the relationship between surrogate endpoints and hard outcomes was informed in the model via risk equations investigating the association between respective risk factors and the incidence of diseases.

Using a closed-cohort approach, the cumulative incidence of complications and rates of events, costs, life-years (LYs) gained, and quality-adjusted LYs (QALYs) gained were calculated. The cohort was defined using a set of average, baseline characteristics, reflective of the target population, to which treatment effects were applied for the duration of treatment. These treatment effects were gradually lost thereafter, according to a regain rate. The model underwent several validations, including three published external validations [47–49].

### Model inputs

#### Transition probabilities and risk equations

Detailed information on the risk equations and transition probabilities used has been provided in earlier publications [47, 48]. Briefly, the incidence of T2D, first-occurring CV-events and recurrent events was predicted using the QDiabetes [50], QRisk3 [51], and Framingham Recurrent CHD [52] risk prediction algorithms, respectively.

The prevalence of sleep apnea was calculated in the model using data from a multicenter cohort, namely the Sleep Heart Health Study [53]. The baseline incidence of colon cancer and its association with BMI were sourced from Body Mass and Colorectal Cancer Risk in the NIH-AARP study [54]. The incidence of post-menopausal breast and endometrial cancers and knee replacement was sourced from two systematic reviews and meta-analyses [55, 56] and a case-control study [57], respectively.

#### Weight-loss and glycemic status maintenance during treatment

The results of STEP 5 were used to inform changes in weight and prediabetes reversal in the second year of treatment. It was done by applying a ratio of change (e.g., ratio of Week 104 weight change to Week 68 weight change) to the change applied in Year 1, as observed at Week 68 in STEP 1 and STEP 2 and reported in the NMA, year by year thereafter, thus, creating a diminishing effect of treatment over time. Further, as data were not available for liraglutide 3.0 mg, orlistat, and NB-32, their maintenance of treatment effect was assumed to be equal to that of semaglutide 2.4 mg.

#### Weight regain and glycemic worsening following cessation of treatment

A return rate of 54% was applied in the first cycle (year) post-treatment cessation, bringing the values of the surrogate clinical efficacy endpoints back to their baselines from the second cycle (year) after treatment cessation (i.e., 100% return rate in 2nd year). These rates were sourced from patients who completed 20-weeks of treatment with semaglutide 2.4 mg and were randomized to switch to placebo for another 48 weeks in STEP 4 [58]. The rate of return to prediabetes was 12.7%, 48 weeks post-treatment switch. Faster and slower catch-up rates were tested in sensitivity analyses, i.e., based on results of STEP 1 Extension [59], and a published independent economic evaluation by the National Institute for Health Research (NIHR) in the UK respectively [60]. In the STEP 1 Extension study, both semaglutide 2.4 mg and D&E were discontinued, and outcomes were observed one year after discontinuation [59]. A natural increase in weight was assumed after the catch-up period, while SBP and lipids were assumed to remain at baseline levels. Glycemic worsening in the cohort without and with diabetes was represented by the transitions from normal glucose or prediabetes to T2D according to QDiabetes [50] and by the UKPDS68 HbA1c trajectory equation [61], respectively. A summary of key modeling assumptions and justifications is provided in Supplementary Table 8.

#### Mortality

Obesity is linked with an increased risk of mortality, particularly from CV diseases and cancers [62, 63]. Overall, obesity is estimated to increase CV- and cancer-related mortality by 4- and 2-fold, respectively. People with severe obesity have a 6- to 12-fold increased all-cause mortality rate [64]. In the analyses conducted, mortality was accounted for by using the general population, all-cause age, and gender annual probabilities of death sourced from Canadian life tables [65, 66]. Life tables were adjusted to exclude deaths due to obesity complications using mortality by cause of death and subtracting those from the all-cause mortality (non-disease-specific mortality [Supplementary Table 9]). The non-disease specific mortality was then adjusted with HR per unit increase in BMI from a large study on the UK Clinical Practice Research Datalink (*N* = 3.6 million adults) [67]. This was done to account for the additional mortality associated with obesity [49], due to causes largely unaccounted for in the model and given a noted underestimation of mortality in the validation [49]. The BMI-dependent HRs were then multiplied with case fatalities for the events modeled (MI, UA, stroke, knee osteoarthritis surgery) and HRs post-ACS, post-stroke, and T2D (Supplementary Table 10). A scenario analysis was conducted whereby weight-loss effect on mortality was excluded thus the BMI-dependent HRs on mortality were removed.

#### Costs

A societal perspective on costs was taken, meaning costs were included regardless of whether they were covered by public or private insurance, or paid out of pocket by patients. Work productivity losses (WPL) were also included in this analysis. The list price of semaglutide 2.4 mg was provided by Novo Nordisk. Other drug prices used in the analysis were sourced from IQVIA Delta Price Advisor are detailed in Supplementary Table 11. The cost of D&E was estimated as an average of the four programs recommended by the Canadian Adult Obesity Clinical Practice Guidelines [68] (Supplementary Table 11), and gym costs were accounted for as costs to private payers. These costs were applied to all treatment arms. The costs of managing weight-related complications, applied either as chronic recurring health state costs, or as one-off costs of events, were sourced from the literature and the Ontario Care Costing Initiative (Supplementary Table 11) [69]. Costs that may be covered by both public and private payers depending on individual coverage (e.g., costs of BP medication), were included only once from the societal perspective. Disease monitoring costs were assumed to consist of four annual visits and two annual blood checks and were applied to all patients in the analyzed cohorts. All costs published prior to 2021 were inflated using the consumer price index to May 2021 (the latest published index as of July 2021) [70].

#### Utilities

The association between BMI and health-related quality of life (QoL), as well as age and gender, was informed via an analysis of 36-Item Short Form Survey (SF-36) data collected in STEP 1 [36]. The results on the SF-36 were mapped to the European QoL 5-Dimensions 3-Level Version (EQ-5D-3L) at the patient level using UK general population tariffs. Then, baseline EQ-5D-3L scores were regressed against baseline BMI, controlling for age, presence of coronary artery disease, prediabetes, hypertension, and smoking status at baseline in STEP 1. The regression was implemented in the model to provide a baseline, complication-free utility dependent on the cohort’s BMI in cycle, age, and sex (using the corresponding regression coefficients, Supplementary Table 12). This regression followed the approach taken by Søltoft et al. whose results had been used in sensitivity analysis [71]. Next, event and health state disutilities were applied, using an additive approach, as the cohort moved across comorbidity health states or experienced events. Disutilities applied to comorbidities and events were sourced from the literature, such that they represented a marginal, complication-specific disutility, thus ensuring the additive approach did not result in double-counting (Supplementary Table 13).

### Analysis

#### Base-case

The base-case analysis was conducted for a mixed population with NGT (26%), prediabetes (46%), and T2D (28%) at baseline and a treatment duration of 2 years in ERs, in line with the maximum duration over which semaglutide 2.4 mg safety and efficacy has been investigated.

#### Deterministic sensitivity analysis (DSA)

Extensive DSA were conducted including upper and lower variations for over 150 model parameters (Supplementary Table 14). These include longer treatment durations (5 and 10 years), baseline cohort characteristics from two real-world studies of liraglutide 3.0 mg use in Canada [72] and semaglutide 2.4 mg use in the US [73], alternate clinical efficacy inputs, disutilities and costs, among others. Results of DSA were presented as tornado plots of the top 15 most influential variables.

#### Scenario analyses

Key structural and methodological uncertainty were further tested in scenario analyses. These included: [1] an analysis without treatment stopping rules for non-responders, in line with a true intention-to-treat efficacy reported in the clinical trials and the NMA, [2] a combined scenario whereby the trial product estimand efficacy was applied for semaglutide 2.4 mg and D&E (anchoring the NMA results for the other comparators to the efficacy estimated for semaglutide 2.4 using the trial product estimand) and separately accounting for the effect of treatment discontinuation via a per cycle discontinuation rate and switch to the D&E arm followed by next-line BaS; and [3] a scenario analysis comparing semaglutide 2.4 mg with liraglutide 3.0 mg using the results of the direct, head-to-head trial, STEP 8 [74]. Structural uncertainty regarding the existence of pharmacological weight-loss effects on complications such as cancers, mortality, major CV-events and knee osteoarthritis surgeries was tested in multiple scenarios and all combined. T2D and sleep apnea were included in all analyses as previously shown to respond to weight-loss pharmacotherapy [12, 14–18]. Finally, scenarios 2,3, and 4 were also run without the stopping rule.

#### Probabilistic sensitivity analyses (PSA)

PSA were conducted with 1000 iterations, given, ICUR convergence was observed after ~300 iterations (Supplementary Fig. 2). The standard error (SE) of the means for all parameters tested in PSA are shown in each corresponding input table (Supplementary Tables 3, 4). The results of the PSA are presented in the cost-effectiveness plane and cost-effectiveness acceptability frontier (CEAF).

## Results

### BMI trajectory

**Fig. 1:**
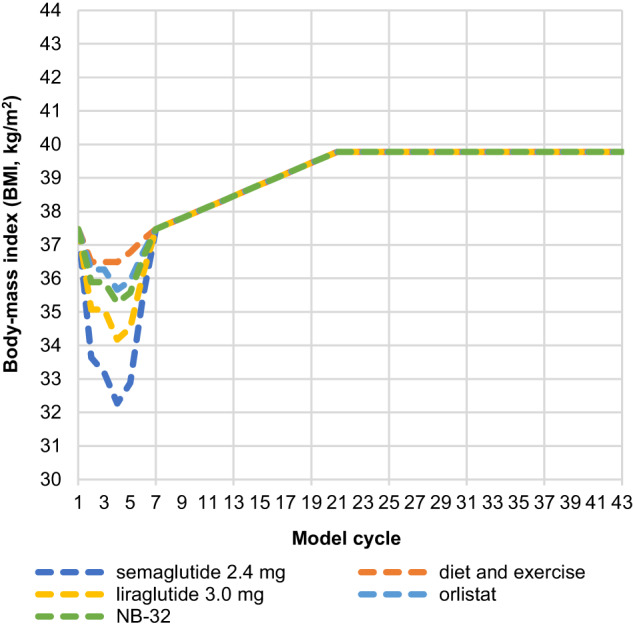
BMI trajectory. BMI body mass index, NB-32 naltrexone 32 mg/bupropion 360 mg.

BMI trajectory presents the changes in BMI observed with each treatment up to cycle 5, the maximum effect being observed with semaglutide 2.4 mg followed by liraglutide 3.0 mg, NB-32, orlistat and D&E. Beyond this time, and up to cycle 7, an assumed rebound in weight upon treatment discontinuation was applied, which brought the BMI trajectory of all arms converging to the starting/baseline BMI. From cycle seven and onwards, the progression depicts a natural increase in BMI (Fig. 1).

### Base case

Table 1 reports the total costs including intervention costs and obesity related disease costs, net impact on total costs, and total QALYs for each modeled intervention. ICERs were reported for each intervention compared to current SoC and incrementally against the next most effective (in terms of QALYs) alternative.

**Table 1:** Base-case cost-effectiveness results (costs are 2021 CAD).

	Intervention Cost*	Obesity disease cost	Total cost	Total QALY	Incr. cost	Incr. QALY	ICUR (vs. next best alternative)	ICUR vs. SoC
Base-case results, societal perspective including WPL								
Diet and exercise	0	208 458	208 458	18.18				
Orlistat	1901	207 551	209 452	18.22	994	0.04	23 479	23 479
NB-32 mg	3336	207 427	210 763	18.23	Ext. dom.	Ext. dom.	Ext. dom.	45 842
Liraglutide 3.0 mg	5839	206 783	212 622	18.27	Ext. dom.	Ext. dom.	Ext. dom.	46 476
Semaglutide 2.4 mg	6737	205 999	212 736	18.32	3284	0.11	31 243	29 014
Results, private payer perspective only								
Diet and exercise	0	144 916	144 916	18.18				
Orlistat	1901	144 558	146 459	18.22	1543	0.04	36 475	36 475
NB-32 mg	3336	144 516	147 852	18.23	Ext. dom.	Ext. dom.	Ext. dom.	58 398
Liraglutide 3.0 mg	5839	144 271	150 110	18.27	Ext. dom.	Ext. dom.	Ext. dom.	57 983
Semaglutide 2.4 mg	6737	144 028	150 765	18.32	4306	0.11	40 966	39 677
Results, public payer perspective only								
Diet and exercise	0	54 944	54 944	18.18				
Orlistat	1901	54 531	56 432	18.22	1488	0.04	35 169	35 169
NB-32 mg	3336	54 476	57 812	18.23	Ext. dom.	Ext. dom.	Ext. dom.	57 024
Liraglutide 3.0 mg	5839	54 202	60 041	18.27	Ext. dom.	Ext. dom.	Ext. dom.	56 883
Semaglutide 2.4 mg	6737	53 813	60 550	18.32	4118	0.11	39 180	38 029
Scenario 1, intention-to-treat analysis (no stopping rule)								
Diet and exercise	0	208 458	208 458	18.18				
Orlistat	3592	206 948	210 540	18.24	2081	0.06	33 585	33 585
NB-32 mg	6307	206 706	213 013	18.25	Ext. dom.	Ext. dom.	Ext. dom.	64 170
Liraglutide 3.0 mg	7740	206 397	214 137	18.27	Dominated	Dominated	Dominated	60 487
Semaglutide 2.4 mg	8169	205 934	214 103	18.33	3563	0.09	40 825	37 819
Scenario 2, cycle discount + trial product estimand + next-line bariatric surgery								
Diet and exercise	0	208 488	208 488	18.18				
Orlistat	1546	207 732	209 277	18.21	789	0.04	20 744	20 744
NB-32 mg	3336	207 653	210 989	18.22	Ext. dom.	Ext. dom.	Ext. dom.	58 146
Liraglutide 3.0 mg	5868	206 988	212 855	18.26	Ext. dom.	Ext. dom.	Ext. dom.	54 742
Semaglutide 2.4 mg	6798	206 227	213 025	18.31	3747	0.10	38 481	33 497
Scenario 3, comparison based on the head-to-head trial STEP 8 [74]								
Liraglutide 3.0 mg	5301	179 907	185 208	19.391				
Semaglutide 2.4 mg	6530	178 407	184 936	19.479	–271	0.09	Dominant	NA
Scenario 4, excluding weight-loss effect on complications and mortality (combined)								
Diet and exercise	0	208 482	208 482	18.16				
Orlistat	1901	207 587	209 488	18.19	1005	0.03	35 723	35 723
NB-32 mg	3335	207 462	210 797	18.19	Ext. dom.	Ext. dom.	Ext. dom.	78 817
Liraglutide 3.0 mg	5837	206 830	212 667	18.21	Ext. dom.	Ext. dom.	Ext. dom.	81 373
Semaglutide 2.4 mg	6734	206 041	212 775	18.24	3287	0.06	58 851	51 102

^*^In the societal perspective, intervention costs are accounted for only once, as either private or public. CAD: Canadian dollars, Dominated: An intervention that is both more costly and less effective than a comparator. A dominated intervention does not offer good value for money and is therefore excluded from the calculation of ICURs. Ext Dom: Extendedly dominated: An intervention that is excluded because an alternative intervention can deliver greater QALY gains for a lower ICUR. ICUR incremental cost-utility ratio, Incr. incremental, NA not applicable, NB-32 naltrexone 32 mg/bupropion 360 mg, QALY quality-adjusted life-years, SoC standard of care, WPL work productivity losses.

From a societal perspective, at a WTP threshold of CAD 50 000 per QALY gained, semaglutide 2.4 mg was the most effective and cost-effective treatment among other weight-management pharmacotherapies approved by Canada Health and recommended by the Canadian Adult Obesity Clinical Practice Guidelines, based on an ICUR of 31 243 per QALY gained versus the next best alternative, i.e., orlistat and an ICUR of 29 014 per QALY gained versus current SoC, i.e., D&E in the base case analysis. NB-32 and liraglutide 3.0 mg were extendedly dominated by semaglutide 2.4 mg as the later delivered higher QALYs (18.32) at lower ICUR (CAD 31 243 per QALY gained) while orlistat had an ICUR of CAD 23 479 per QALY gained versus D&E but was associated with lower QALY gains (0.04) versus semaglutide 2.4 mg (0.11). When taking a restricted private or public payer perspective, NB-32 and liraglutide 3.0 mg remained extendedly dominated by semaglutide 2.4 mg and the latter was projected to remain the preferred intervention resulting in an ICUR of ~CAD 40 000 CAD per QALY gained versus orlistat under each of the two perspectives.

**Fig. 2:**
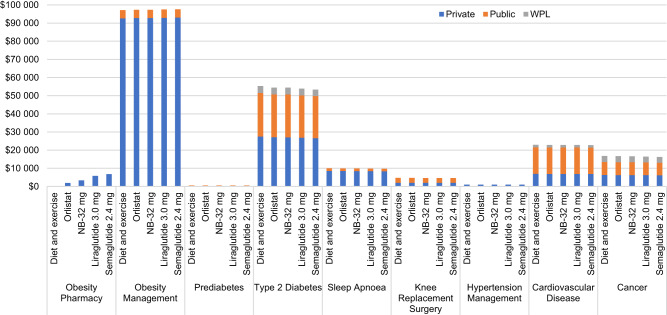
Costs breakdown. NB-32 naltrexone 32 mg/bupropion 360 mg, WPL work productivity losses.

Patients on semaglutide 2.4 mg followed by liraglutide 3.0 mg, NB-32, orlistat, and D&E, can be expected to spend less time with obesity related complications, and the highest contribution to this came from the avoidance or delay of T2D, prediabetes reversal, and reduction in sleep apnea prevalence with weight-loss. Moreover, treatment with semaglutide 2.4 mg also resulted in higher, albeit marginally, QALYs compared with all other treatments (Supplementary Table 15). Over time, these clinical results translate into cost reductions from treating weight-related diseases, which partially offset the higher drug acquisition costs with semaglutide 2.4 mg. The breakdown of cost results showed that the biggest contributor to cost offsets with semaglutide 2.4 mg were costs associated with delayed T2D and cancer, followed by CV risk from a societal perspective and costs associated with delayed T2D from a private and public payer only perspective (Fig. 2).

### Deterministic sensitivity analysis

**Fig. 3:**
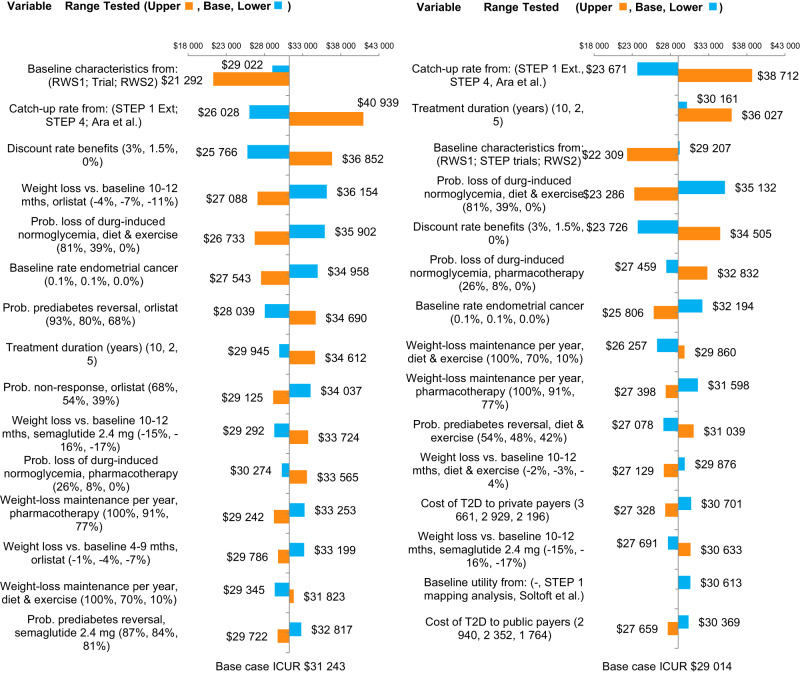
One-way sensitivity analyses. Tornado plots, (**a**) semaglutide 2.4 mg vs orlistat (**b**) semaglutide 2.4 mg vs diet and exercise. Source: RWS1 Wharton et al. Canada-cohort [72], RWS2 Ghush et al US-cohort T2D type 2 diabetes [73]. Ext. extension, ICUR incremental cost-utility ratio, RWS real world studies, T2D type 2 diabetes.

The results of DSA are presented in Fig. 3 in the form of tornado plots of the 15 most influential single parameters. The top three major drivers with the highest impact on the cost-effectiveness results for semaglutide 2.4 mg versus next best alternative, i.e. orlistat were (1) using different sets of baseline cohort characteristics from real-world studies of liraglutide 3.0 mg use in Canada [72] and semaglutide 2.4 mg use in the US [73], both of which decreased the ICUR; (2) using a faster catch-up rate post-treatment period from STEP 1 Extension, which increased the ICUR, and a slower catch-up from Ara et al. [58–60] which decreased it; and (3) discounting rates applied to benefits which both increased and decreased the ICUR. In all analyses, the ICUR remained below the WTP of CAD 50 000 per QALY.

The sensitivity analyses with the highest impact on the cost-effectiveness results for semaglutide 2.4 versus the current SoC were (1) a faster catch-up rate post-treatment period from STEP 1 Extension [59], (2) longer treatment durations (up to 10 years), whereby incremental treatment costs with semaglutide 2.4 mg increased more rapidly than the incremental benefits, and (3) the use of baseline cohort characteristics from the two real-world studies [72] and [73]. In all cases, ICURs remained below the WTP of CAD 50 000 per QALY.

Longer treatment durations, using a faster catch-up rate post-treatment period from STEP 1 Extension [59], weight-loss applied in cycle 4 (10-12 months) and discount rate for benefits of 1.5% and 0% were the drivers with the highest impact on the cost-effectiveness results for orlistat, NB-32 and liraglutide 3.0 mg when compared to SoC, i.e., D&E (Supplementary Fig. 3).

### Scenarios

Semaglutide 2.4 mg remained the most cost-effective treatment among weight-management pharmacotherapies in scenarios 1 and 2, i.e., continuation of treatment in non-responders, and the combined analysis of cycle discontinuation plus trial product estimand efficacy and next-line BaS, respectively. ICURs for scenarios 1 and 2 were CAD 40 825 and CAD 38 481 per QALY gained versus orlistat, and CAD 37 819 and CAD 33 497 per QALY gained versus D&E, respectively. In the comparison based on the head-to-head trial, STEP 8 (scenario 3), semaglutide 2.4 mg was projected to be a dominant treatment alternative to liraglutide 3.0 mg as it resulted in both lower incremental cost (CAD -271) and higher QALYs (0.09) (Table 1).

In scenarios whereby the modeled weight-loss effect on cancer, mortality, CV disease and knee osteoarthritis surgery, were taken individually, semaglutide 2.4 mg remained the preferred intervention with ICURs below CAD 50 000 per QALY (Supplementary Table 16). Yet, in a scenario whereby the effect of weight-loss on all four complications combined was excluded (scenario 4) Table 1, the resulting ICUR for semaglutide 2.4 mg was estimated to be 58 851 per QALY gained, greater than the WTP threshold of CAD 50 000 per QALY. Hence, if the modeled weight-loss benefits on cancer, mortality, CV, or osteoarthritis surgeries are excluded simultaneously, orlistat emerges as the best value for money alternative compared to current SoC, with an ICUR of CAD 35 723 per QALY gained.

In additional scenario analyses combining continuation of treatment independent of weight-loss response (i.e., continuation in non-responders) with scenarios 2, 3 and 4 respectively, the ICUR for semaglutide 2.4 mg was marginally above the cost-effectiveness WTP at an ICUR of CAD 50 038 per QALY vs. orlistat, which emerged as the best value for money (scenario 9, Supplementary Table 16), remained dominant compared to liraglutide 3.0 mg (scenario 10, Supplementary Table 16), and, consistently with results obtained in scenario 4, orlistat was also the best value for money when the effects of weight-loss on cancer, mortality, CVD and surgeries for knee osteoarthritis were excluded simultaneously.

### Probabilistic sensitivity analysis

**Fig. 4:**
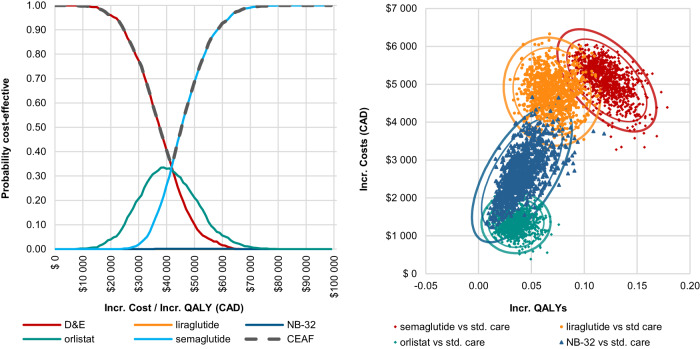
Probabilistic analyses. **a** Cost-effectiveness plane and (**b**) cost-effectiveness acceptability frontier. CEAF cost-effectiveness acceptability frontier, CAD Canadian dollar, Incr incremental, ICUR incremental cost-utility ratio, NB-32 naltrexone 32 mg/bupropion 360 mg, QALY quality-adjusted life-year, std standard.

The cost-effectiveness plane displayed 100% of ICURs in the northeast (NE) quadrant, indicating little uncertainty with regard to the existence of additional costs and additional QALY benefits for orlistat, NB-32, liraglutide 3.0 mg, and semaglutide 2.4 mg versus SoC, respectively (Fig. 4a).

The CEAF indicated that at the defined WTP threshold of CAD 50 000 per QALY gained, the probability of semaglutide 2.4 mg, orlistat, liraglutide 3.0 mg, NB-32, and D&E to be cost-effective was 70%, 20%, 0%, 0% and 10%, respectively. It was also observed that at varying WTP threshold levels, at any value at or above CAD 42 000 per QALY gained, semaglutide 2.4 mg was the most likely economically preferred intervention (Fig. 4b).

## Discussion

In this study, semaglutide 2.4 mg was found to be a cost-effective treatment alternative compared with D&E alone (current SoC), or with orlistat (next best alternative), and extendedly dominated other pharmacotherapies (NB-32, or liraglutide 3.0 mg) in adults living with obesity or with overweight and ≥1 weight-related comorbidity. The analyses conducted rely on surrogate effects of weight loss and glycemic reductions leading to delays in the incidences of weight-related diseases and costs. The modeled associations were informed via large, observational, cohort studies, e.g., QDiabetes [50], QRisk3 [51], Framingham Recurrent CHD [52] or meta meta-analyses of observational studies [55, 56]. The analysis further assumed that weight loss had an instantaneous effect on incidences, and that the effect did not depend on prior weight or time spent living with obesity.

The use of surrogate endpoints in the context of HTA is increasingly common [75, 76], and certain HTA agencies are seeking to develop methodological guidelines for their use in cost-effectiveness analyses [77]. In their evaluation of semaglutide 2.4 mg, the UK National Institute for Health and Care Excellence (NICE) highlighted the uncertainties related to the use of risk equations in the context of weight-management interventions yet acknowledged the use of risk equations as the only method currently available to predict long-term outcomes in obesity [78, 79]. NICE recognized the benefits of semaglutide 2.4 mg in delaying the onset of diabetes as both striking and important and recommended its reimbursement in people living with obesity and ≥1 weight-related comorbidity.

In turn, the Canadian Agency for Drugs and Technologies in Health (CADTH), emphasized on the fact that, other than glycemic control, STEP trials did not showcase sufficient evidence to support an association between short-term weight-loss with semaglutide 2.4 mg and improvement in weight-related conditions such as CV diseases, mortality, or cancers [80]. The agency further challenged the assumption of 2-year treatment duration, citing the likelihood of it having a meaningful impact on comorbidities and highlighting the uncertainty with regards to semaglutide 2.4 mg cost-effectiveness under longer treatment durations. In our analyses, we explored the impact on cost-effectiveness for treatment durations of 5 and 10 years and found that these did not change the study conclusions. Regarding the modeled effects on weight-related conditions, we believe that the CADTH perspective fails to recognize the importance of sustained, clinically relevant levels of weight loss and improvement in glycemic control. For e.g., data from the Swedish Obese Subjects (SOS) trial showed reductions in overall mortality (HR 0.71; 95% CI 0.52; 0.92), female cancers’ incidences (HR = 0.58; *P* = 0.0008), MI (HR = 0.71; *P* = 0.02), and stroke (HR = 0.66; *P* = 0.008) and several fold increases in diabetes remission 20 years following BaS [10]. The mean weight reductions observed with semaglutide 2.4 mg are greater than those observed with other pharmacotherapies, yet not as high as those observed with BaS. Thus, in the analyses conducted, we explored the uncertainty related to the realization of cancer, mortality, and CV disease risk reductions on cost-effectiveness, and noted that their individual exclusion did not change the analysis conclusions. However, when these scenarios were combined, semaglutide 2.4 mg was no longer the preferred intervention and orlistat became the most cost-effective treatment alternative to the current SoC. This scenario may be considered extreme and unlikely due to the emerging evidence on semaglutide 2.4 mg benefits on hard, patient and economically relevant endpoints.

Indeed, lower doses of semaglutide, 0.5 mg and 1.0 mg, have been shown to reduce the risk of non-fatal MI and non-fatal stroke in a cardiovascular study in people with type 2 diabetes and high-risk of CV disease. It was postulated that the sustained and clinically meaningful reductions in HbA1c, body weight, and SBP all contributed to the observed reduction in CV risk with semaglutide [25]. Oral semaglutide was also shown to reduce CV-deaths and non-fatal strokes in a similar population [81]. Neither of these studies were conducted in people living with obesity. Yet, there are several ongoing trials investigating the effects of semaglutide 2.4 mg on weight-related comorbidities, including the SELECT study, in patients with overweight or obesity who have established CV diseases [82], two heart failure trials in patients with overweight or obesity with and without T2D [83, 84], and a trial in knee osteoarthritis [85]. The results of these studies will partially address the current evidence gaps regarding the effects of semaglutide 2.4 mg on comorbidity-related outcomes.

The generalizability of the patient population investigated in our analyses, from the STEP 1 and STEP 2 trials [36, 37], was tested in two scenario analyses whereby the cohort’s baseline characteristics were sourced from two real-world studies of semaglutide 2.4 mg and liraglutide 3.0 mg [72, 73]. These scenarios demonstrated that the baseline characteristics used closely reflect a real-life population and that treating populations with glycemic impairment, before the onset of T2D, can be expected to result in higher cost-effectiveness for semaglutide 2.4 mg, which is not surprising given its impact on glycemic control.

Uncertainty in the current analyses is also noted regarding the comparative effectiveness versus other pharmacotherapies, as there are limited direct head-to-head trials. A potential source of bias was identified in the methods used to address missing data in the NMA. Particularly, in studies of NB-32 and orlistat, the last observation carried forward (LOCF) method was used, while a multiple imputations method approach was used in the STEP trials. As explained by Wharton et al., the underlying assumption of the LOCF is violated in weight-loss studies, and as such, this may have biased results in favor of orlistat and NB-32 [86].

In addition to uncertainties, the results of our study should be interpreted considering certain limitations. One of these was the generalizability of the risk equations to the Canadian population. As neither of the risk algorithms used were estimated in Canadian populations, it is uncertain whether the overall level of risk applied in the model, as well as the reduction in risk, is fully generalizable. Based on a recent systematic literature review [87], there are presently no risk equations specific to the Canadian population. Furthermore, to the best of our knowledge, there are no marked differences in risk factors leading to CVD or T2D between the Canadian and UK populations [88]. Thus, we believe that the UK populations underlying the QRisk3, QDiabetes, and UKPDS82 are good proxies. Another limitation is regarding the cost and QoL parameters for microvascular complications related to T2D, whereby a single cost and disutility were applied to the proportion of the cohort with T2D throughout the analysis time horizon. As new proportions of the cohort enter the T2D state each cycle, it was not possible to include different costs or QoL for T2D (linked to, e.g., severity or duration of the disease) without including additional health states.

## Conclusion

Based on the base-case analysis, semaglutide 2.4 mg was cost-effective in comparison to both orlistat and D&E at a WTP threshold of CAD 50 000 per QALY gained, and extendedly dominated NB-32 and liraglutide 3.0 mg in adults with obesity or with overweight and ≥1 weight-related condition from a societal perspective in Canada. Limiting the perspective to either the public or the private payer perspective increased the ICUR to around CAD 40 000 per QALY gained versus orlistat but maintained semaglutide 2.4 mg as the preferred intervention. Almost all sensitivity and scenario analyses results were aligned with the base-case analyses. The exception were the scenarios in which the weight-related effect on the studied cancers, mortality, CV disease and knee osteoarthritis surgery were simultaneously removed. In these scenarios, orlistat emerged as the most cost-effective treatment alternative to SoC at a WTP of CAD 50 000 per QALY gained. Orlistat was also the most cost-effective alternative when treatment continuation was assumed to take place in all non-responders, the ICUR for semaglutide 2.4 mg being just marginally above the WTP threshold. However, in reality, the ICUR for semaglutide 2.4 may be expected to fall in between the base-case and this scenario, thus making semaglutide 2.4 mg the preferred intervention, given, most patients may be expected to discontinue treatment in case of non-response, in alignment with Canadian clinical practice guidelines.

### Supplementary information

Suppl material Cost-effectiveness of WM Pharmacotherpaies in Canada

## Data Availability

There is a restriction applied to the data that support the findings of this study; therefore, they are not publicly available. Data are, however, available from the authors upon contract agreement and with the permission of Novo Nordisk. Please contact the corresponding author, M Luckevich (MLVH@novonordisk.com), to request the data from this study.
