# Supplementary material for: Cost-effectiveness of weight-management pharmacotherapies in Canada: a societal perspective
Source: Int J Obes (Lond). 2024 Jan 31;48(5):683–93. doi: 10.1038/s41366-024-01467-w (PMC11058048; doi:10.1038/s41366-024-01467-w)
Supplement: Supplementary file 1 — Suppl material Cost-effectiveness of WM Pharmacotherpaies in Canada [file 41366_2024_1467_MOESM1_ESM.docx]

**Supplementary material**

**Cost-Effectiveness of Weight Management Pharmacotherapies in Canada: A Societal Perspective**

Authors: Anamaria-Vera Olivieri, Sergey Muratov, Sara Larsen, Maria Luckevich, Katalina Chan, Mark Lamotte and David CW Lau

**List of figures and tables**

[Supplementary Fig 1. Core Obesity Model structure. 2](#_Toc153044894)

[Supplementary Fig 2. Incr. costs and incr. QALY convergence graphs for semaglutide 2.4 mg vs diet and exercise 3](#_Toc153044895)

[Supplementary Fig 3. Tornado diagrams. 4](#_Toc153044896)

[Supplementary Table 1. Cohort characteristics at baseline, weighted for glycemic status: normal glucose tolerance + prediabetes + T2D. 8](#_Toc153044900)

[Supplementary Table 2. Percent changes in weight, systolic blood pressure, and prediabetes reversal vs. baseline by glycemic status subgroup in ER population. 9](#_Toc153044901)

[Supplementary Table 3. Percent change in total and HDL cholesterol vs. baseline for all treatments, by glycemic status subgroup in ER population. 11](#_Toc153044902)

[Supplementary Table 4. Percent changes in weight, systolic blood pressure, T-chol, HDL and prediabetes reversal vs. baseline trial product estimand in STEP studies. 13](#_Toc153044903)

[Supplementary Table 5. Percent treatment discontinuation applied in scenario with trial product estimand, including non-responder discontinuation. 14](#_Toc153044904)

[Supplementary Table 6. Percent changes in weight, systolic blood pressure, and prediabetes reversal vs. baseline by glycemic status subgroup in FAS population 15](#_Toc153044905)

[Supplementary Table 7. Probability (%) of adverse events. 17](#_Toc153044906)

[Supplementary Table 8. Summary of base-case and scenario analysis settings and assumptions. 18](#_Toc153044907)

[Supplementary Table 9. Annual probability of death by age and gender in Canada, 2019. 25](#_Toc153044908)

[Supplementary Table 10. Disease specific probabilities of death applied in model. 28](#_Toc153044909)

[Supplementary Table 11. Direct and indirect costs included in model (CAD, 2021). 29](#_Toc153044910)

[Supplementary Table 12. Model used for prediction of body mass index-dependent utilities. 39](#_Toc153044911)

[Supplementary Table 13. Disutilities associated with health states and acute events. 39](#_Toc153044912)

[Supplementary Table 14. Upper and lower values tested in univariate sensitivity analyses on model parameters. 41](#_Toc153044913)

[Supplementary Table 15. Breakdown of clinical results 47](#_Toc153044914)

[Supplementary Table 16. Scenario analyses (costs are 2021 CAD). 48](#_Toc153044915)

Supplementary Fig 1. Core Obesity Model structure.


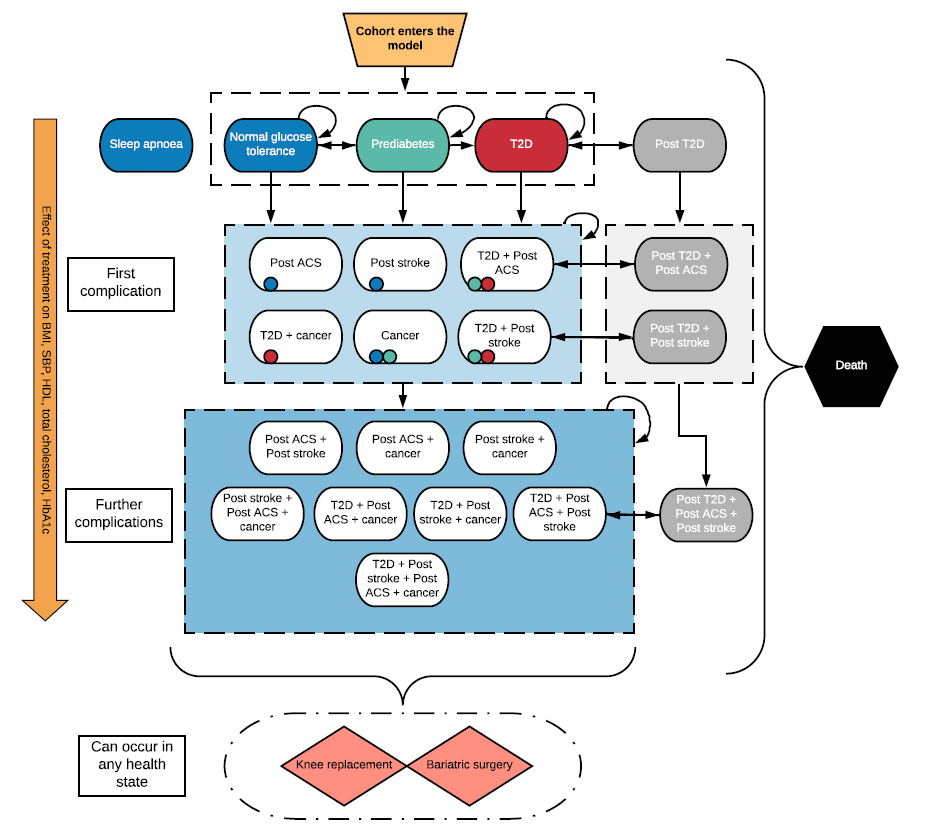


Reproduced/adapted from Lopes 2021 [[1](#_ENREF_1)] with permission from Wiley. ACS acute coronary syndrome, BMI body mass index, HbA_1c_ glycated hemoglobin, HDL high-density lipoprotein, SBP systolic blood pressure, T2D type 2 diabetes.

Supplementary Fig 2. Incr. costs and incr. QALY convergence graphs for semaglutide 2.4 mg vs diet and exercise


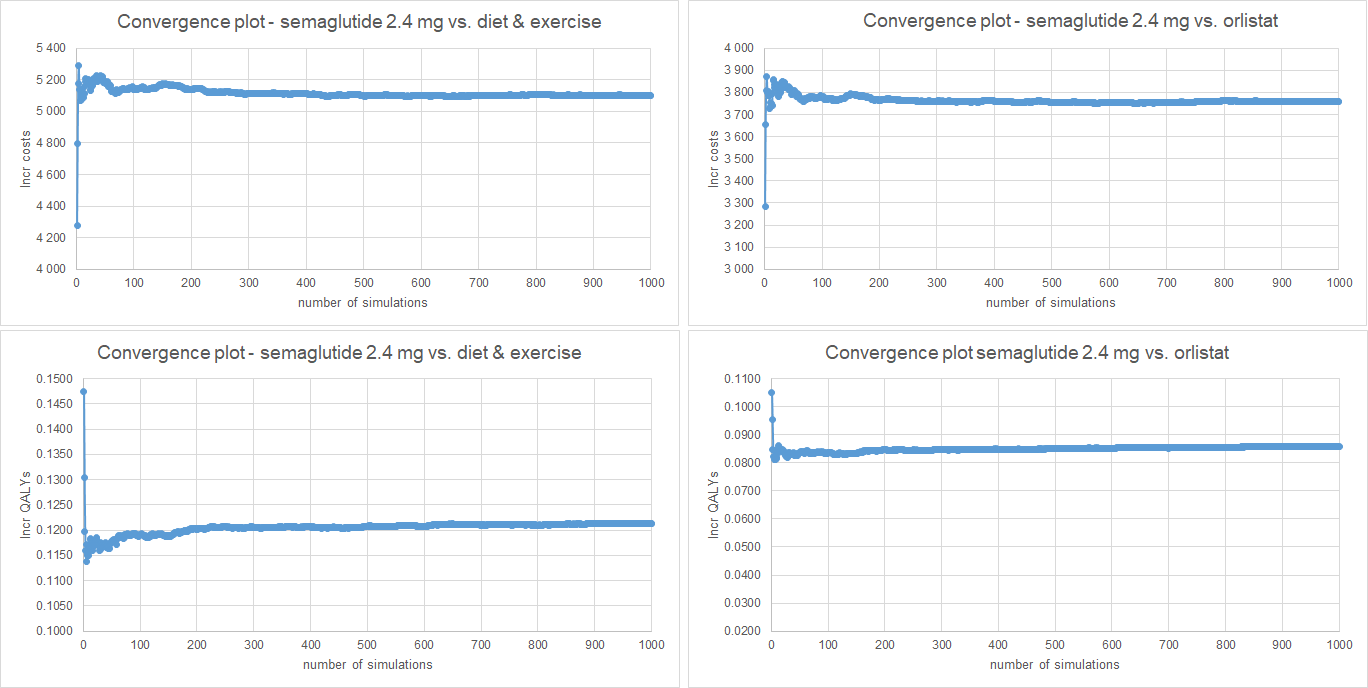


Supplementary Fig 3. Tornado diagrams.

1. Orlistat vs diet and exercise


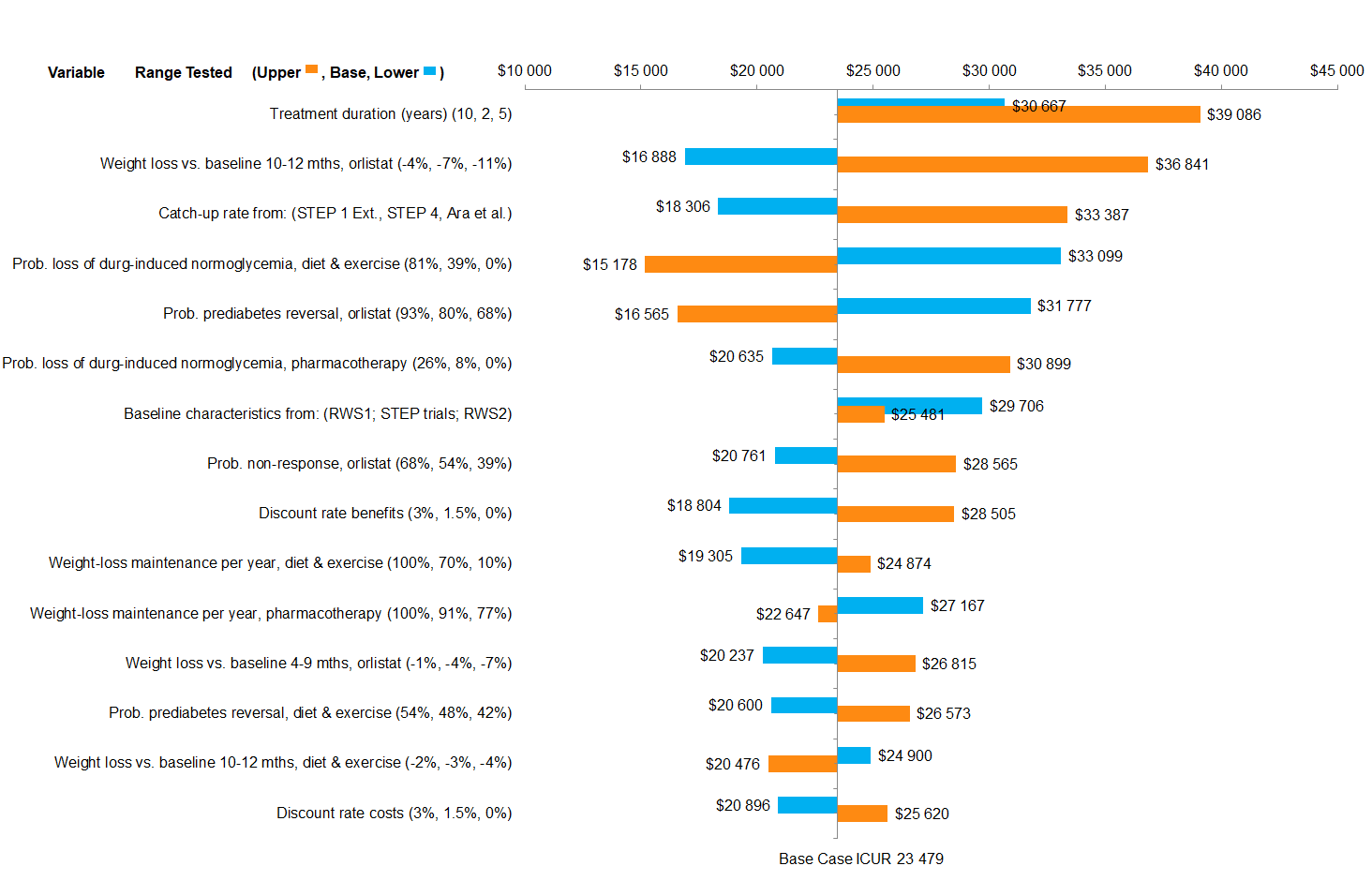


1. NB-32 vs diet and exercise


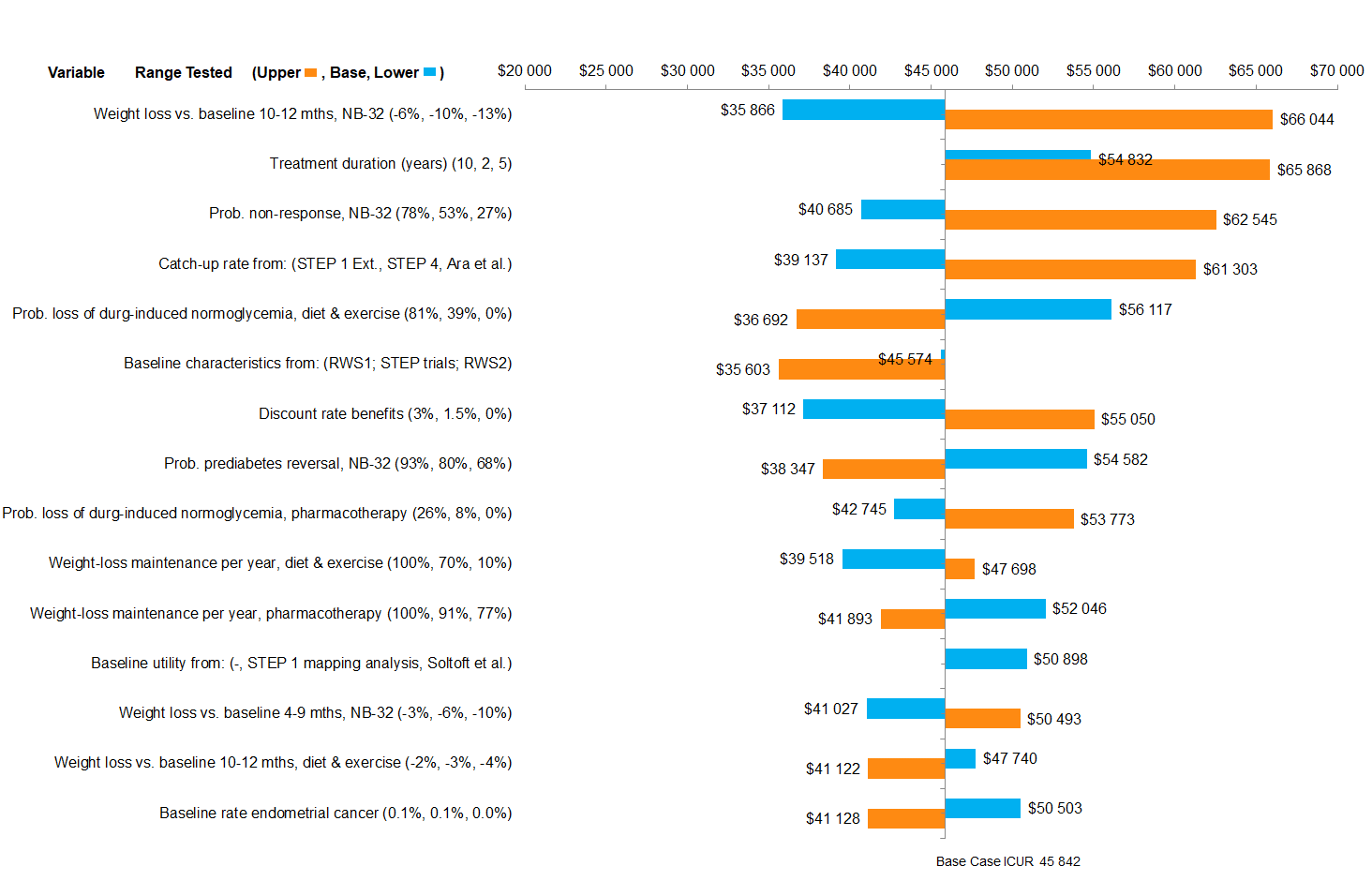


1. Liraglutide 3.0 mg vs. diet and exercise


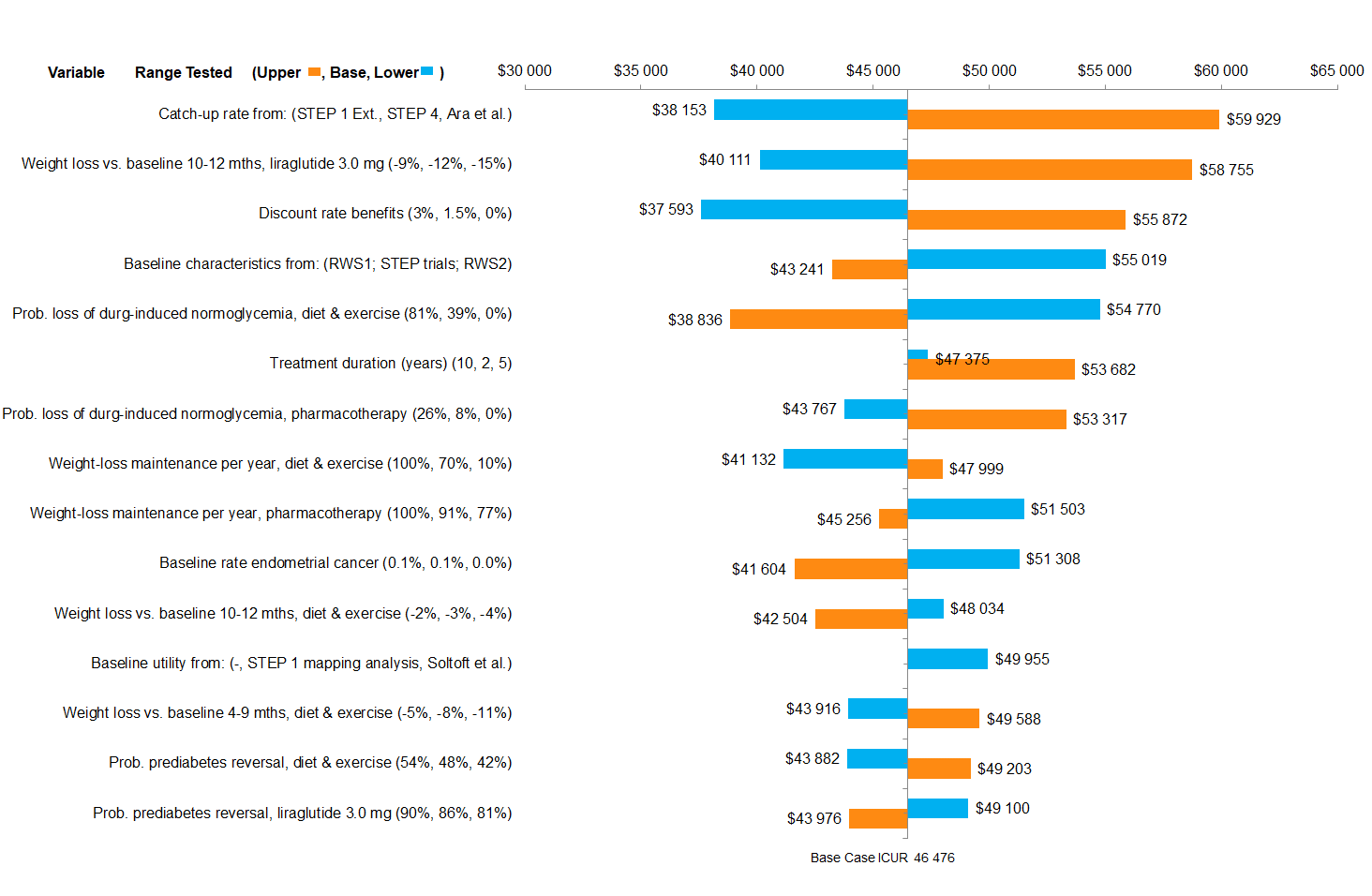


Supplementary Table 1. Cohort characteristics at baseline, weighted for glycemic status: normal glucose tolerance + prediabetes + T2D.

|  | Weighted mean | Normal glucose tolerance | | Prediabetes | | | T2D | | | STEP 8 cohort |
| --- | --- | --- | --- | --- | --- | --- | --- | --- | --- | --- |
|  |  | **Mean** | **SEM** | **Mean** | **SEM** | **Source** | **Mean** | **SEM** | **Source** | **Mean** |
| Age (years) | 49.6 | 45.0 | 0.4 | 49.0 | 0.4 | [[2](#_ENREF_2)] | 55.0 | 0.3 | [[2](#_ENREF_2)] | 48.7 |
| Body mass index (kg/m^2^) | 37.5 | 37.3 | 0.2 | 38.5 | 0.2 | [[2](#_ENREF_2)] | 35.9 | 0.2 | [[2](#_ENREF_2)] | 37.1 |
| Height (m) | 1.66 | 1.67 | 0.0 | 1.66 | 0.0 | [[2](#_ENREF_2)] | 1.67 | 0.0 | [[2](#_ENREF_2)] | 1.7 |
| SBP (mmHg) | 127.8 | 125.0 | 0.4 | 128.0 | 0.5 | [[2](#_ENREF_2)] | 130.0 | 0.4 | [[2](#_ENREF_2)] | 125.8 |
| Total cholesterol (mg/dL) | 189.0 | 194.1 | 1.1 | 194.4 | 1.4 | [[2](#_ENREF_2)] | 175.2 | 1.1 | [[2](#_ENREF_2)] | 190.8 |
| HDL cholesterol (mg/dL) | 49.1 | 52.3 | 0.4 | 49.4 | 0.4 | [[2](#_ENREF_2)] | 45.5 | 0.3 | [[2](#_ENREF_2)] | 54.3 |
| HbA_1c_ (%) | NA | NA | NA | NA | NA | NA | 8.1 | 0.0 | [[3](#_ENREF_3)] | NA |
| T2D duration (years) | NA | NA | NA | NA | NA | NA | 8.0 | 0.2 | [[3](#_ENREF_3)] | NA |
| Triglycerides (mg/dL) | 154.4 | 138.6 | 3.4 | 148.2 | 2.7 | [[2](#_ENREF_2)] | 179.7 | 3.1 | [[2](#_ENREF_2)] | 126.1 |
| Smokers (%) | 36.6 | 25.8 | 1.3 | 39.6 | 1.7 | [[2](#_ENREF_2)] | 41.9 | 1.4 | [[2](#_ENREF_2)] | 30.8 |
| Females (%) | 66.8 | 76.9 | 1.3 | 70.4 | 1.6 | [[2](#_ENREF_2)] | 51.2 | 1.4 | [[2](#_ENREF_2)] | 78.7 |

*HbA_1c_* glycated hemoglobin; *HDL* high-density lipoprotein, *SBP* systolic blood pressure, *T2D* type 2 diabetes, *SEM* standard error of the mean, *NA* not applicable.

Supplementary Table 2. Percent changes in weight, systolic blood pressure, and prediabetes reversal vs. baseline by glycemic status subgroup in ER population.

|  | Semaglutide 2.4 mg ER | | D&E FAS | | Liraglutide 3.0 mg ER | | Orlistat ER | | NB-32 mg ER | |
| --- | --- | --- | --- | --- | --- | --- | --- | --- | --- | --- |
|  | Mean | SEM | Mean | SEM | Mean | SEM | Mean | SEM | Mean | SEM |
| **Weight-loss (%)** | | | | | | | | | | |
| Subgroup: normal glucose tolerance at baseline | | | | | | | | | | |
| Cycles 2 & 3 | **-13.7** | **0.2** | **−2.6** | **0.3** | **−5.5** | **0.7** | **-3.9** | **1.2** | **−6.2** | **1.3** |
| Cycle 4 | **-18.6** | **0.4** | **−2.4** | **0.5** | **−10.5** | **0.7** | **-8.8** | **1.2** | **−11.2** | **1.3** |
| Subgroup: prediabetes at baseline | | | | | | | | | | |
| Cycles 2 & 3 | **-12.2** | **0.2** | **−2.6** | **0.3** | **−9.2** | **0.8** | **-2.4** | **1.2** | **−4.8** | **1.3** |
| Cycle 4 | **-16.1** | **0.4** | **−2.4** | **0.5** | **−13.0** | **0.8** | **-6.3** | **1.2** | **−8.7** | **1.3** |
| Subgroup: type 2 diabetes at baseline | | | | | | | | | | |
| Cycles 2 & 3 | **-11.3** | **0.2** | **−2.6** | **0.2** | **−8.9** | **3.6** | **-6.4** | **2.9** | **−8.0** | **3.2** |
| Cycle 4 | **-12.8** | **0.4** | **−3.1** | **0.3** | **−10.5** | **3.6** | **-7.9** | **2.9** | **−9.5** | **3.2** |
| Scenario STEP 8, normal glucose tolerance or prediabetes at baseline | | | | | | | | | | |
| Cycles 2 & 3 | -11.4 | 0.3 | NA | NA | -9.3 | 0.3 | NA | NA | NA | NA |
| Cycle 4 | -19.0 | 0.5 | NA | NA | -8.8 | 0.3 | NA | NA | NA | NA |
| **Ratio of weight-loss maintenance (if <1 weight regain)** | | | | | | | | | | |
| Cycles 5+ | **0.91** | **NA** | **0.70** | **NA** | **0.91** | **NA** | **0.91** | **NA** | **0.91** | **NA** |
| **Systolic blood-pressure decrease (mmHg)** | | | | | | | | | | |
| Subgroup: normal glucose tolerance at baseline | | | | | | | | | | |
| Cycles 2 & 3 | **-6.5** | **0.5** | **−0.3** | **0.6** | **−3.1** | **1.0** | **-6.5** | **0.0** | **0.0** | **2.4** |
| Cycle 4 | **-7.8** | **0.5** | **−0.8** | **0.6** | **−4.4** | **1.0** | **-7.8** | **0.0** | **−1.2** | **2.4** |
| Subgroup: prediabetes at baseline | | | | | | | | | | |
| Cycles 2 & 3 | **-5.8** | **0.5** | **−0.3** | **0.7** | **−4.5** | **1.0** | **-5.8** | **0.0** | **0.7** | **2.4** |
| Cycle 4 | **-6.4** | **0.6** | **−1.2** | **0.7** | **−5.1** | **1.0** | **-6.4** | **0.0** | **0.2** | **2.4** |
| Subgroup: type 2 diabetes at baseline | | | | | | | | | | |
| Cycles 2 & 3 | **-6.8** | **0.7** | **−0.1** | **0.6** | **−5.7** | **1.6** | **-5.0** | **1.6** | **−2.2** | **1.6** |
| Cycle 4 | **-5.8** | **0.8** | **0.0** | **0.6** | **−4.7** | **1.6** | **-4.1** | **1.6** | **−1.3** | **1.6** |
| Scenario STEP 8, normal glucose tolerance or prediabetes at baseline | | | | | | | | | | |
| Cycles 2 & 3 | -8.1 | 0.21 | NA | NA | -8.8 | 0.25 | NA | NA | NA | NA |
| Cycle 4 | -7.5 | 0.19 | NA | NA | -5.5 | 0.16 | NA | NA | NA | NA |
| **Ratio SBP change (if <1 SBP increases, if >1 SBP decreases)** | | | | | | | | | | |
| Cycle 5 | **1.00** | **NA** | **1.31** | **NA** | **1.00** | **NA** | **1.00** | **NA** | **1.00** | **NA** |
| **Prediabetes reversal (%), applied in the prediabetes at baseline subgroup only** | | | | | | | | | | |
| Subgroup: prediabetes at baseline | | | | | | | | | | |
| Cycle 2 | **84.1** | **1.5** | **47.8** | **3.1** | **85.5** | **2.5** | **80.3** | **6.3** | **80.3** | **6.3** |
| Scenario STEP 8 with prediabetes at baseline | | | | | | | | | | |
| Cycle 2 | 81.6 | 5.7 | NA | NA | 63.2 | 5.7 | NA | NA | NA | NA |
| **Probability of return to prediabetes** | | | | | | | | | | |
| Cycles 5+ | **8.0** | **9.4** | **38.9** | **21.2** | **8.0** | **9.4** | **8.0** | **9.4** | **8.0** | **9.4** |
| **Probability early responder (weight change of 5% or more vs. baseline, all patients)** | | | | | | | | | | |
| Cycle 2 | **78.6** | **NA** | **23.8** | **NA** | **68.2** | **NA** | **46.1** | **NA** | **46.9** | **NA** |
| Scenario STEP 8 | | | | | | | | | | |
| Cycle 2 | 24.6 | 7.7 | NA | NA | 38.6 | 7.0 | NA | NA | NA | NA |

Note: values applied in the model base-case cost-effectiveness analyses are highlighted in bold, SBP is expressed as absolute mean change, mmHg. D&E diet and exercise, ER early responder, FAS full analysis set, NA not applicable, NB-32 naltrexone 32/bupropion 360, NGT normal glucose tolerance, SEM standard error of mean, T2D type 2 diabetes.

Supplementary Table 3. Percent change in total and HDL cholesterol vs. baseline for all treatments, by glycemic status subgroup in ER population.

| Change (%) | Semaglutide 2.4 mg ER | | Diet and exercise FAS | | Liraglutide 3.0 mg ER | | Orlistat ER | | Naltrexone 32 mg /bupropion ER | |
| --- | --- | --- | --- | --- | --- | --- | --- | --- | --- | --- |
|  | Mean | SEM | Mean | SEM | Mean | SEM | Mean | SEM | Mean | SEM |
| **Total cholesterol** | | | | | | | | | | |
| Subgroup: NGT at baseline | | | | | | | | | | |
| Cycle 2 | **-16.2** | **0.2** | **0.2** | **0.0** | **-12.6** | **1.9** | **-16.2** | **0.0** | **−16.2** | **0.0** |
| Subgroup: prediabetes at baseline | | | | | | | | | | |
| Cycle 2 | **-14.5** | **0.2** | **2.4** | **0.0** | **−10.9** | **1.9** | **-14.5** | **0.0** | **−14.5** | **0.0** |
| Cycle 3 & 4 | **-7.0** | **0.1** | **2.4** | **0.0** | **−3.2** | **2.0** | **-7.0** | **0.0** | **−7.0** | **0.0** |
| Subgroup: T2D at baseline | | | | | | | | | | |
| Cycle 2 | **-15.6** | **0.2** | **−0.2** | **0.0** | **−20.4** | **2.8** | **-15.6** | **0.0** | **−15.6** | **0.0** |
| Cycle 3 & 4 | **-3.9** | **0.1** | **−0.4** | **0.0** | **−8.9** | **3.0** | **-3.9** | **0.0** | **−3.9** | **0.0** |
| Scenario STEP 8 | | | | | | | | | | |
| Cycle 2 | -17.3 | 0.44 | NA | NA | -5.8 | 0.16 | NA | NA | NA | NA |
| Cycle 3&4 | -15.4 | 0.40 | NA | NA | 1.9 | 0.05 | NA | NA | NA | NA |
| **HDL cholesterol** | | | | | | | | | | |
| Subgroup: NGT at baseline | | | | | | | | | | |
| Cycle 2 | **-5.1** | **0.1** | **−0.9** | **0.0** | **−6.1** | **0.6** | **-5.1** | **0.0** | **−5.1** | **0.0** |
| Cycle 3 & 4 | **2.8** | **0.0** | **0.6** | **0.0** | **1.8** | **0.7** | **2.8** | **0.0** | **2.8** | **0.0** |
| Subgroup: prediabetes at baseline | | | | | | | | | | |
| Cycle 2 | **-4.0** | **0.0** | **−0.9** | **0.0** | **−4.4** | **0.6** | **-4.0** | **0.0** | **−4.0** | **0.0** |
| Cycle 3 & 4 | **3.2** | **0.0** | **1.1** | **0.0** | **2.7** | **0.7** | **3.2** | **0.0** | **3.2** | **0.0** |
| Subgroup: T2D at baseline | | | | | | | | | | |
| Cycle 2 | **-1.2** | **0.0** | **0.2** | **0.0** | **−1.2** | **0.8** | **-1.2** | **0.0** | **−1.2** | **0.0** |
| Cycle 3 & 4 | **4.4** | **0.1** | **1.6** | **0.0** | **4.4** | **0.9** | **4.4** | **0.0** | **4.4** | **0.0** |
| Scenario STEP 8 | | | | | | | | | | |
| Cycle 2 | -4.3 | 0.11 | NA | NA | -1.4 | 0.04 | NA | NA | NA | NA |
| Cycle 3 & 4 | 0.5 | 0.01 | NA | NA | 1.9 | 0.05 | NA | NA | NA | NA |

Note: values applied in the model base-case cost-effectiveness analyses are highlighted in bold.
ER early responder, FAS full analysis set; HDL high-density lipoprotein, NGT normal glucose tolerance, SEM standard error of mean, T2D type 2 diabetes.

Supplementary Table 4. Percent changes in weight, systolic blood pressure, T-chol, HDL and prediabetes reversal vs. baseline trial product estimand in STEP studies.

|  | Semaglutide 2.4 mg ER | | Diet & exercise FAS | | Liraglutide 3.0 mg ER | | Orlistat ER | | NB-32 mg ER | |
| --- | --- | --- | --- | --- | --- | --- | --- | --- | --- | --- |
|  | Mean | SEM | Mean | SEM | Mean | SEM | Mean | SEM | Mean | SEM |
| **Weight loss (%)** | | | | | | | | | | |
| Subgroup: normal glucose tolerance at baseline | | | | | | | | | | |
| Cycle 2 & 3 | -14.0 | 0.2 | -2.8 | NA | -5.8 | NA | -4.1 | 1.3 | -6.5 | 1.3 |
| Cycle 4 | -19.7 | 0.4 | -2.4 | NA | -11.5 | NA | -9.9 | 1.3 | -12.2 | 1.3 |
| Subgroup: prediabetes at baseline | | | | | | | | | | |
| Cycle 2 & 3 | -12.3 | 0.2 | -2.8 | NA | -9.3 | NA | -2.5 | 1.2 | -4.9 | NA |
| Cycle 4 | -17.0 | 0.4 | -2.4 | NA | -13.9 | NA | -7.2 | 1.2 | -9.5 | NA |
| Subgroup: type 2 diabetes at baseline | | | | | | | | | | |
| Cycle 2 & 3 | -11.4 | 0.3 | -2.7 | NA | -9.0 | NA | -6.5 | 2.9 | -8.1 | NA |
| Cycle 4 | -13.2 | 0.4 | -3.1 | NA | -10.9 | NA | -8.4 | 2.9 | -9.9 | NA |
| Ratio of weight-loss maintenance (i.e., weight regain) | | | | | | | | | | |
| Cycles 5+ | 0.96 | NA | 0.71 | NA | 0.96 | NA | 0.96 | NA | 0.96 | NA |
| **Systolic blood pressure decrease (%)** | | | | | | | | | | |
| Subgroup: normal glucose tolerance at baseline | | | | | | | | | | |
| Cycle 2 & 3 | -6.86 | 0.48 | −1.0 | NA | −3.5 | NA | -6.86 | 0.0 | -0.3 | NA |
| Cycle 4 | -8.39 | 0.51 | −1.1 | NA | −5.0 | NA | -8.39 | 0.0 | −1.9 | NA |
| Subgroup: prediabetes at baseline | | | | | | | | | | |
| Cycle 2 & 3 | -6.06 | 0.55 | −1.0 | NA | -4.8 | NA | -6.06 | 0.00 | 0.5 | NA |
| Cycle 4 | -6.68 | 0.59 | −1.1 | NA | -5.4 | NA | -6.68 | 0.00 | -0.1 | NA |
| Subgroup: type 2 diabetes at baseline | | | | | | | | | | |
| Cycle 2 & 3 | -6.94 | 0.70 | −0.0 | NA | −5.9 | NA | -5.22 | 1.61 | −2.4 | NA |
| Cycle 4 | -6.39 | 0.80 | −0.5 | NA | −5.3 | NA | -4.67 | 1.61 | −1.9 | NA |
| Ratio SBP change (if <1 SBP increases, if >1 SBP decreases) | | | | | | | | | | |
| Cycle 5 | 0.86 | NA | 1.11 | NA | 0.86 | NA | 0.86 | NA | 0.86 | NA |
| **Prediabetes reversal (%), applied in the prediabetes at baseline subgroup only** | | | | | | | | | | |
| Subgroup: prediabetes at baseline | | | | | | | | | | |
| Cycle 2 | 88.1 | 1.5 | 47.8 | 3.1 | 85.5 | 2.5 | 80.3 | 6.3 | 80.3 | 6.3 |
| Probability of return to prediabetes | | | | | | | | | | |
| Cycles 5+ | 11.3 | 9.8 | 41.2 | 32.1 | 11.3 | 9.8 | 11.3 | 9.8 | 11.3 | 9.8 |

Note: values applied in the model base-case cost-effectiveness analyses are highlighted in bold; SBP is expressed as absolute mean change, mmHg. *ER* early responder, *FAS* full analysis set, *NA* not applicable, *NB* naltrexone 32/bupropion 360, *NGT* normal glucose tolerance, *SEM* standard error of mean, *T2D*, type 2 diabetes.

Supplementary Table 5. Percent treatment discontinuation applied in scenario with trial product estimand, including non-responder discontinuation.

|  | **Semaglutide 2.4 mg** | **D&E** | **Liraglutide 3.0 mg** | **Orlistat** | **NB-32 mg** |
| --- | --- | --- | --- | --- | --- |
| Cycle 1 | 0.0 | 0 | 3.8 | 3.8 | 3.8 |
| Cycle 2 | 5.8 | 0 | 36.9 | 59.0 | 58.3 |
| Cycle 3 | 29.9 | 0 | 39.5 | 61.6 | 60.8 |
| Cycle 4 | 31.8 | 0 | 42.1 | 64.1 | 63.4 |
| Cycle 5 | 36.6 | 0 | 45.9 | 68.0 | 67.3 |
| Cycle 6 | 53.2 | 0 | 60.0 | 82.1 | 81.4 |

From cycle 3 onwards for semaglutide 2.4 mg and from cycle 2 onwards for liraglutide 3.0 mg, orlistat and NB-32 discontinuation is inclusive of non-responder discontinuation. *D&E* diet and exercise*, NB-32* naltrexone 32/bupropion 360

Supplementary Table 6. Percent changes in weight, systolic blood pressure, and prediabetes reversal vs. baseline by glycemic status subgroup in FAS population

|  | Semaglutide 2.4 mg FAS | | D&E FAS | | Liraglutide 3.0 mg FAS | | Orlistat FAS | | NB-32 mg FAS | |
| --- | --- | --- | --- | --- | --- | --- | --- | --- | --- | --- |
|  | Mean | SEM | Mean | SEM | Mean | SEM | Mean | SEM | Mean | SEM |
| **Weight-loss (%)** | | | | | | | | | | |
| Subgroup: normal glucose tolerance at baseline | | | | | | | | | | |
| Cycles 2 & 3 | −11.8 | 0.2 | −2.6 | 0.3 | -3.7 | 0.7 | −2.0 | 1.2 | -4.4 | 1.3 |
| Cycle 4 | −15.9 | 0.3 | −2.4 | 0.5 | -7.7 | 0.7 | −6.1 | 1.2 | -8.4 | 1.3 |
| Subgroup: prediabetes at baseline | | | | | | | | | | |
| Cycles 2 & 3 | −10.4 | 0.2 | −2.6 | 0.3 | -7.4 | 0.8 | −0.6 | 1.2 | -3.0 | 1.3 |
| Cycle 4 | −13.7 | 0.4 | −2.4 | 0.5 | -10.6 | 0.8 | −3.9 | 1.2 | -6.2 | 1.3 |
| Subgroup: type 2 diabetes at baseline | | | | | | | | | | |
| Cycles 2 & 3 | −8.4 | 0.2 | −2.6 | 0.2 | -6.1 | 3.6 | −3.5 | 2.9 | -5.1 | 3.2 |
| Cycle 4 | −9.6 | 0.3 | −3.1 | 0.3 | -7.3 | 3.6 | −4.8 | 2.9 | -6.4 | 3.2 |
| Scenario STEP 8, normal glucose tolerance or prediabetes at baseline | | | | | | | | | | |
| Cycles 2 & 3 | -9.28 | NA | NA | NA | -9.32 | NA | NA | NA | NA | NA |
| Cycle 4 | -15.78 | NA | NA | NA | -8.84 | NA | NA | NA | NA | NA |
| **Ratio of weight-loss maintenance (if <1 weight regain)** | | | | | | | | | | |
| Cycles 5+ | 0.91 | NA | 0.70 | NA | 0.91 | NA | 0.91 | NA | 0.91 | NA |
| **Systolic blood-pressure decrease (mmHg)** | | | | | | | | | | |
| Subgroup: normal glucose tolerance at baseline | | | | | | | | | | |
| Cycles 2 & 3 | −5.5 | 0.4 | −0.3 | 0.6 | -2.2 | 1.0 | −5.5 | 0.0 | 1.0 | 2.4 |
| Cycle 4 | −6.4 | 0.4 | −0.8 | 0.6 | -3.1 | 1.0 | −6.4 | 0.0 | 0.1 | 2.4 |
| Subgroup: prediabetes at baseline | | | | | | | | | | |
| Cycles 2 & 3 | −5.0 | 0.5 | −0.3 | 0.7 | -3.7 | 1.0 | −5.0 | 0.0 | 1.6 | 2.4 |
| Cycle 4 | −5.8 | 0.5 | −1.2 | 0.7 | -4.5 | 1.0 | −5.8 | 0.0 | 0.7 | 2.4 |
| Subgroup: type 2 diabetes at baseline | | | | | | | | | | |
| Cycles 2 & 3 | −4.6 | 0.6 | −0.1 | 0.6 | -3.6 | 1.6 | −2.9 | 1.6 | -0.1 | 1.6 |
| Cycle 4 | −3.9 | 0.6 | 0.0 | 0.6 | -2.9 | 1.6 | −2.2 | 1.6 | 0.6 | 1.6 |
| Scenario STEP 8, normal glucose tolerance or prediabetes at baseline | | | | | | | | | | |
| Cycles 2 & 3 | -6.07 | NA | NA | NA | -8.81 | NA | NA | NA | NA | NA |
| Cycle 4 | -5.69 | NA | NA | NA | -5.52 | NA | NA | NA | NA | NA |
| **Ratio SBP change (if <1 SBP increases, if >1 SBP decreases)** | | | | | | | | | | |
| Cycle 5 | 1.00 | NA | 1.31 | NA | 1.00 | NA | 1.00 | 1.00 | NA | NA |
| **Prediabetes reversal (%), applied in the prediabetes at baseline subgroup only** | | | | | | | | | | |
| Subgroup: prediabetes at baseline | | | | | | | | | | |
| Cycle 2 | 84.1 | 1.5 | 47.8 | 3.1 | 80.9 | 3.1 | 74.5 | 7.4 | 74.5 | 7.4 |
| Scenario STEP 8 with prediabetes at baseline | | | | | | | | | | |
| Cycle 2 | 81.6 | NA | NA | NA | 63.2 | NA | NA | NA | NA | NA |
| Probability of return to prediabetes | | | | | | | | | | |
| Cycles 5+ | 10.9 | 10.3 | 38.9 | 21.2 | 10.9 | 10.3 | 10.9 | 10.3 | 10.9 | 10.3 |

Note: SBP is expressed as absolute mean change, mmHg. D&E diet and exercise, ER early responder, FAS full analysis set, NA not applicable, NB-32 naltrexone 32/bupropion 360, NGT normal glucose tolerance, SEM standard error of mean, T2D type 2 diabetes.

Supplementary Table 7. Probability (%) of adverse events.

|  | **Semaglutide 2.4 mg** | **D&E** | **Liraglutide 3.0 mg** | **Orlistat** | **NB-32 mg** |
| --- | --- | --- | --- | --- | --- |
| Severe gastrointestinal | | | | | |
| NGT & prediabetes at baseline | 4.8 | 0.0 | 4.6 | 6.4 | 3.1 |
| Type 2 diabetes at baseline | 2.3 | 0.0 | 19.7 | 3.7 | 3.1 |
| Severe hypoglycemia | | | | | |
| NGT & prediabetes at baseline | 0.0 | 0.0 | 0.1 | NA | NA |
| Type 2 diabetes at baseline | 0.2 | 0.0 | 4.9 | NA | NA |
| Non-severe hypoglycemia | | | | | |
| NGT & prediabetes at baseline | 0.8 | 0.0 | 15.6 | NA | NA |
| Type 2 diabetes at baseline | 44.6 | 0.0 | 58.1 | NA | NA |

*Note:* probabilities shown are applied per 1-year cycles and are adjusted in the model for shorter (3-months) cycles. *D&E* diet and exercise*, NB-32* naltrexone 32/bupropion 360, *NA* not applicable, *NGT* normal glucose tolerance

Supplementary Table 8. Summary of base-case and scenario analysis settings and assumptions.

| Model parameter | Base-case | Scenarios/ sensitivity analyses | Justification/assumption |
| --- | --- | --- | --- |
| Target population | Adults with BMI 27-30 kg/m^2^ and one or more weight-related comorbidity including hypertension, dyslipidemia, OSA, cardiovascular disease or T2D, or with BMI ≥30 kg/m^2^ | Using two real-world cohorts of people treated with liraglutide in Canada (from Wharton et al [[4](#_ENREF_4)]) and treated for obesity with semaglutide injection 2.4 mg in the US (Ghusn et al [[5](#_ENREF_5)]) | In line with the Canadian product monograph [[6](#_ENREF_6)] |
| Time horizon | 40 years | Not conducted | Forty years, considered sufficiently long to capture all health and costs benefits for 1 year of treatment in a population with starting age of 50 years. Additionally, most risk equations informing the prediction of CV events have 2, 5 or maximum 10 years of follow-up data; thus, applying the prediction of these risk equations over longer periods may be associated with uncertainty |
| Discount rate costs and benefits | 1.5% | 3% and 0% | In accordance with the CADTH economic guidelines |
| Perspective | Societal (private + public + patient + work productivity losses) | Public only, then private only | As per Canadian health economic guidelines |
| Treatment effects |  |  | Assumption: modelling assumes that the effect on health depends on current BMI, not past BMI. This assumption is inherent to the current data available on the association between BMI and risk of complications, and has been used in other obesity modelling analyses, such as that of Boyers et al. https://doi.org/10.1038/s41366-  021-00849-8. |
| Estimand | Treatment policy | Trial product | The treatment policy estimand (intention-to-treat) included data from all subjects regardless of premature trial product discontinuation and/or use of rescue medication |
| Include early responder analysis (i.e., stop treatment if ≥5% weight loss not achieved after 12 weeks on maintenance dose) | Yes, for all pharmacotherapies | No for all treatments | As per Canada product monographs for semaglutide injection 2.4 mg and comparators, clinical guidelines for diet and exercise |
| Treatment duration | 2 years | 5 and 10 years | Maximum treatment duration was chosen in line with the duration over which semaglutide injection 2.4 mg efficacy was studied in the target population. Longer treatment durations of 5 and 10 years are explored in scenarios, using results of STEP 5 applied year-to-year |
| Include per-cycle discontinuation before end of fixed treatment period | No, in line with ITT analysis | Yes, in scenario with trial product estimand |  |
| Responders discontinue to: | Diet and exercise | Not conducted |  |
| Include disutility and costs of treatment-related adverse events | Yes, for severe gastrointestinal (all treatments), and severe and non-severe hypoglycemia (semaglutide injection 2.4 mg and liraglutide 3.0 mg) | Exclude |  |
| Weight regains and return to prediabetes after treatment stop (catch-up rate) | Weight return rate from STEP 4 [[7](#_ENREF_7)]: 54% in first year, 100% in second year  return to prediabetes: 12.7% per year | Return rate from STEP 1 extension [[8](#_ENREF_8)]: weight, SBP, lipids: year 1 64%, years 2-4 (exponential extrapolation): 87-95-100%;  return to prediabetes: year 1: 34%, Years 2-3 (linear extrapolation): 69-100%  Return rate from Ara et al [[9](#_ENREF_9)]: weight, SBP, lipids: 33% year 1, 67% year 2, 100% year 3 |  |
| Weight at the end of catch-up period: | Return to baseline value | Not conducted |  |
| Natural weight increase after treatment stop: | Ara et al [[9](#_ENREF_9)], (CPRD) 0.145 kg/m^2^ and 0.175 kg/m2 per cycle (year) in males and females, respectively, up to a maximum age of 68 years. | Not conducted |  |
| Bariatric surgery (BaS) |  |  |  |
| Include BaS | Not included | Yes, as next-line treatment | A BMI threshold was run to show the impact of including it in a scenario, however in the Canadian context a BMI threshold of 35 kg/m^2^ lacks face validity as BaS is only available to <1% of people eligible (and between BMI 35-40 they also must have a severe comorbidity), and that historically only people with much higher BMIs have been getting access to this procedure in Canada [[10](#_ENREF_10), [11](#_ENREF_11)].  The efficacy of bariatric surgery is applied in the year when it occurs and maintained throughout the analysis (conservatively, for simplicity). Weight loss with three types of bariatric surgeries is informed as observed at one year in The Swedish Obese Subjects Study [[12](#_ENREF_12)] and weighted by the proportion each type of surgery is performed in Canada using CIHI (2014) data [[13](#_ENREF_13)]. Efficacy on SBP, lipids and HbA1c are source from Demssie et al [[14](#_ENREF_14)].   \|  \| Proportion \| Weight loss \| \| --- \| --- \| --- \| \| Gastric bypass \| 53.0% \| -32.0% \| \| Lap banding \| 19.0% \| -20.0% \| \| Sleeve gastrectomy \| 28.0% \| -25.0% \| |
| Incidence (%) of BaS per year | Not included | 0.37% [[13](#_ENREF_13)] |  |
| Utilities | | | |
| Method to derive baseline utility: | Association analysis of baseline BMI and SF-36 mapped to EQ-5D-3L utilities in STEP 1 [[15](#_ENREF_15)] | Based on Soltoft et al [[16](#_ENREF_16)] | Provides a measure of QoL in relation to BMI gender and age, free of impact of other obesity complications |
| Complications included in the model | | | |
| ACS | Yes, predicted incidence based on QRisk3 during NGT, and UKPDS82 for first and recurrent events respectively in T2D | Setting BMI equal to baseline BMI in all cycles, thus removing weight-loss effects on CV endpoints | Based on evidence on the increased risk associated with obesity as well as demonstrated risk reductions associated with weight loss [[17-19](#_ENREF_17)] |
| Prediabetes | Yes, included as baseline prevalence based on trial characteristics | Not conducted | Assumption: when experiencing a CV event, for reasons of simplicity (keeping number of health states reasonable and not increasing run time) prediabetes patients transition directly to T2D + post-ACS or T2D + post-stroke, i.e., there is no health state to encompass prediabetes + post-ACS or + post-stroke. An analysis was performed for Wegovy^®^ appraisal to NICE, showing that the impact of having the cohort transition from prediabetes to post-ACS or post-stroke had a minor effect on the ICUR, which increased by 3.4% [[20](#_ENREF_20)].  In turn, there is no risk of CV disease applied to patients residing in prediabetes state additional to what is applied in the NGT/no comorbidity state (contrary to what is observed in real-life whereby patients with prediabetes are at increased risk of CV disease). This has the effect of counterbalancing the potential effect of overestimating secondary CV risks after transition from prediabetes to T2D + ACS or stroke, as mentioned above. |
| T2D | Yes, predicted incidence based on QDiabetes risk model | Not conducted | Based on evidence, increased risk associated with obesity as well as demonstrated risk reductions associated with weight loss [[17](#_ENREF_17), [19](#_ENREF_19), [21](#_ENREF_21)]  Assumption: T2D microvascular complications are not included as distinct health states. Rather, for a proportion of the cohort residing in a T2D health state, higher costs apply, reflective of possible microvascular complications. More details on this are provided in Supplementary Table 10 |
| Stroke (including TIA) | Yes, predicted incidence based on same risk models as for ACS | Setting BMI equal to baseline BMI in all cycles, thus removing weight-loss effects on CV endpoints | Based on evidence on the increased risk associated with obesity as well as demonstrated risk reductions associated with weight loss [[17-19](#_ENREF_17)] |
| Sleep apnea | Yes, as prevalence at baseline based on the Sleep Heart Study [[22](#_ENREF_22)] | Not conducted | Based on evidence on the increased risk associated with obesity as well as demonstrated risk reductions associated with weight loss [[17](#_ENREF_17), [19](#_ENREF_19), [22](#_ENREF_22)]  Assumption: sleep apnea is included as a prevalent complication at baseline; thus, it is not mutually exclusive to other states, i.e., it will co-occur with any other of the complications considered. This is a simplifying assumption (i.e., does not affect transitions to or from other states), as having it as a health state would have doubled the number of modeled health states. Furthermore, the presence of sleep apnea was assumed not to impact mortality nor to increase the risk of CV events, to avoid adding substantial complexity to the model. As such, the model will underestimate effects for the more efficacious treatments. |
| Knee replacement | Yes, based on the large (n=911 cases and *n* = 5 578 controls) study of Wendelboe et al [[23](#_ENREF_23)] | Setting BMI equal to baseline BMI in all cycles, thus removing weight-loss effects on knee replacement | Based on evidence on the increased risk associated with obesity as well as demonstrated risk reductions associated with weight loss [[17](#_ENREF_17), [19](#_ENREF_19), [23](#_ENREF_23)]  Assumption: Osteoarthritis is represented in the model as an event, rather than a health state. This is because osteoarthritis per se is not expected to be associated with high costs, while these may occur at the moment of a knee replacement surgery. The surgery event can happen at any time. An annual probability of knee replacement is applied. Despite osteoarthritis being a chronic condition, it is not accounted for as a health state as this would have tripled the number of health states considered. To compensate for the loss in QoL, a once-off disutility is applied with the knee replacement event, and this is multiplied by 3 to account for the years spent in less health before surgery. Pre-surgery costs from 1 year before surgery are also accounted for within the cost of knee replacement surgery |
| Colon cancer | Yes, based on the large study of Adams et al (*n* = ~500 000 subjects of whom 3 300 developed colorectal cancer over the study period) [[24](#_ENREF_24)] | Setting BMI equal to baseline BMI in all cycles, thus removing weight-loss effects on colon cancer | Based on empirical evidence of the increased risk associated with obesity as well as demonstrated risk reductions associated with weight loss [[17](#_ENREF_17), [19](#_ENREF_19), [24](#_ENREF_24)] |
| Post-menopausal endometrial cancer | Yes, based on a systematic literature review reporting incidence of cancer in association with overweight | Setting BMI equal to baseline BMI in all cycles, thus removing weight-loss effects on cancer | Based on evidence on the increased risk associated with obesity as well as demonstrated risk reductions associated with weight loss [[17](#_ENREF_17), [19](#_ENREF_19), [25](#_ENREF_25), [26](#_ENREF_26)] |
| Post-menopausal breast cancer | Yes, based on a systematic literature review reporting incidence of cancer in association with overweight | Setting BMI equal to baseline BMI in all cycles, thus removing weight-loss effects on cancer | Based on evidence on the increased risk associated with obesity as well as demonstrated risk reductions associated with weight loss [[17](#_ENREF_17), [19](#_ENREF_19), [25](#_ENREF_25), [26](#_ENREF_26)] |
| BMI-adjustment applied to mortality: | Disease mortality and BMI (CPRD) | Setting BMI equal to baseline BMI in all cycles, thus removing weight-loss effects | Other approaches have been shown to underestimate mortality with increasing BMI [[1](#_ENREF_1), [27](#_ENREF_27)] |

*AARP* American Association of Retired Persons, *ACS* acute coronary syndrome, *BMI* body mass index, *CADTH* Canadian Agency for Drugs and Technologies in Health, *CPRD* Clinical Practice Research Datalink, *CV* cardiovascular, *EQ-5D-3L* European Quality of Life 5 Dimensions 3 Level Version, *NB-32* naltrexone 32 mg/bupropion, *NGT* normal glucose tolerance, *NIH* National Institutes of Health, *OSA*, obstructive sleep apnea, *QoL* quality of life, *SF-36* Short-Form Survey 36, *SBP* systolic blood pressure, *T2D* type 2 diabetes, *TIA* transient ischemic attack, *UKPDS* UK Prospective Diabetes Study.

Supplementary Table 9. Annual probability of death by age and gender in Canada, 2019.

| Age (years) | Probability that a person aged exactly x will die before reaching age (x +1), non-disease specific | | All-cause mortality, excluding mortality causes in the general population already accounted for in the model | |
| --- | --- | --- | --- | --- |
|  | Males | Females | Males | Females |
| 50 | 0.00290 | 0.00185 | 0.00224 | 0.00156 |
| 51 | 0.00315 | 0.00201 | 0.00244 | 0.00170 |
| 52 | 0.00343 | 0.00218 | 0.00265 | 0.00184 |
| 53 | 0.00374 | 0.00237 | 0.00289 | 0.00200 |
| 54 | 0.00408 | 0.00258 | 0.00315 | 0.00218 |
| 55 | 0.00445 | 0.00282 | 0.00338 | 0.00239 |
| 56 | 0.00486 | 0.00307 | 0.00369 | 0.00260 |
| 57 | 0.00531 | 0.00336 | 0.00403 | 0.00284 |
| 58 | 0.00580 | 0.00367 | 0.00440 | 0.00310 |
| 59 | 0.00635 | 0.00402 | 0.00482 | 0.00340 |
| 60 | 0.00695 | 0.00440 | 0.00519 | 0.00363 |
| 61 | 0.00761 | 0.00483 | 0.00569 | 0.00399 |
| 62 | 0.00834 | 0.00530 | 0.00623 | 0.00438 |
| 63 | 0.00915 | 0.00582 | 0.00684 | 0.00481 |
| 64 | 0.01004 | 0.00641 | 0.00750 | 0.00529 |
| 65 | 0.01103 | 0.00705 | 0.00822 | 0.00575 |
| 66 | 0.01212 | 0.00778 | 0.00904 | 0.00635 |
| 67 | 0.01333 | 0.00858 | 0.00994 | 0.00700 |
| 68 | 0.01467 | 0.00948 | 0.01094 | 0.00774 |
| 69 | 0.01615 | 0.01048 | 0.01204 | 0.00855 |
| 70 | 0.01780 | 0.01160 | 0.01339 | 0.00934 |
| 71 | 0.01963 | 0.01285 | 0.01476 | 0.01035 |
| 72 | 0.02166 | 0.01426 | 0.01629 | 0.01148 |
| 73 | 0.02392 | 0.01583 | 0.01799 | 0.01275 |
| 74 | 0.02643 | 0.01759 | 0.01988 | 0.01416 |
| 75 | 0.02922 | 0.01957 | 0.02214 | 0.01566 |
| 76 | 0.03233 | 0.02180 | 0.02450 | 0.01744 |
| 77 | 0.03579 | 0.02430 | 0.02712 | 0.01944 |
| 78 | 0.03966 | 0.02712 | 0.03005 | 0.02170 |
| 79 | 0.04397 | 0.03029 | 0.03332 | 0.02423 |
| 80 | 0.04878 | 0.03387 | 0.03691 | 0.02664 |
| 81 | 0.05415 | 0.03792 | 0.04098 | 0.02982 |
| 82 | 0.06015 | 0.04249 | 0.04552 | 0.03342 |
| 83 | 0.06687 | 0.04765 | 0.05060 | 0.03748 |
| 84 | 0.07439 | 0.05351 | 0.05630 | 0.04209 |
| 85 | 0.08280 | 0.06014 | 0.06332 | 0.04667 |
| 86 | 0.09223 | 0.06766 | 0.07054 | 0.05250 |
| 87 | 0.10281 | 0.07620 | 0.07863 | 0.05913 |
| 88 | 0.11467 | 0.08591 | 0.08770 | 0.06667 |
| 89 | 0.12799 | 0.09696 | 0.09788 | 0.07524 |
| 90 | 0.14295 | 0.10953 | 0.11016 | 0.08510 |
| 91 | 0.15935 | 0.12353 | 0.12280 | 0.09597 |
| 92 | 0.17682 | 0.13868 | 0.13626 | 0.10774 |
| 93 | 0.19532 | 0.15499 | 0.15051 | 0.12042 |
| 94 | 0.21478 | 0.17245 | 0.16551 | 0.13398 |
| 95 | 0.23618 | 0.19231 | 0.18200 | 0.14941 |
| 96 | 0.25691 | 0.21202 | 0.19798 | 0.16472 |
| 97 | 0.27830 | 0.23277 | 0.21446 | 0.18085 |
| 98 | 0.30015 | 0.25441 | 0.23130 | 0.19766 |
| 99 | 0.32230 | 0.27678 | 0.24837 | 0.21504 |
| 100 | 0.34455 | 0.29968 | 0.26551 | 0.23283 |
| 101 | 0.36670 | 0.32291 | 0.28258 | 0.25088 |
| 102 | 0.38855 | 0.34624 | 0.29942 | 0.26900 |
| 103 | 0.40993 | 0.36945 | 0.31589 | 0.28704 |
| 104 | 0.43066 | 0.39231 | 0.33187 | 0.30480 |
| 105 | 0.45060 | 0.41461 | 0.34723 | 0.32212 |
| 106 | 0.46963 | 0.43616 | 0.36190 | 0.33887 |
| 107 | 0.48765 | 0.45680 | 0.37579 | 0.35490 |
| 108 | 0.50458 | 0.47640 | 0.38883 | 0.37013 |
| 109 | 0.52040 | 0.49486 | 0.40102 | 0.38447 |
| 110 | 1.0* | 1.0* | 0.77060 | 0.77693 |

Source: Based on all-cause and cause-specific general population mortality, Statistics Canada 2019 [[28](#_ENREF_28)]. *imputed value

Supplementary Table 10. Disease specific probabilities of death applied in model.

|  | **Females** | **Males** | **Source** |
| --- | --- | --- | --- |
| Case fatality MI (% per event) | 0.30 | 0.32 | [[29](#_ENREF_29)] |
| Case fatality angina (% per event) | 0.30 | 0.32 | Assumed equal to MI |
| Case fatality stroke (% per event) | 0.25 | 0.17 | [[29](#_ENREF_29)] |
| Post-ACS (HR applied to general mortality) | 1.30 | 1.30 | [[30](#_ENREF_30)] |
| Post-stroke (HR applied to general mortality) | 2.00 | 2.00 | [[31](#_ENREF_31)] |
| Colorectal cancer (% in year of onset) | 0.301 | 0.301 | [[32](#_ENREF_32)] |
| Breast cancer (% in year of onset) | 0.041 | NA | [[32](#_ENREF_32)] |
| Endometrial cancer (% in year of onset) | 0.105 | NA | [[32](#_ENREF_32)] |
| Cancer (% in years 2+ from onset) | 0.0403 | 0.0403 | [[32](#_ENREF_32)] |

*ACS* acute coronary syndrome, *HR* hazard ratio, *MI* myocardial infarction.

Supplementary Table 11. Direct and indirect costs included in model (CAD, 2021).

| Treatment costs | Inflated (2021) | Value in reference | Cost year | Description and reference |
| --- | --- | --- | --- | --- |
| **Obesity pharmacy cost** | | | | |
| Semaglutide 2.4 mg | **$4 653** | NA | 2021 | Price per pack (4 weekly injections): $356.73  Number of days per year = 365.25  Total cost per year: $356.73/4*365.25/7 = $4 653 |
| Liraglutide 3.0 mg daily - 1st year (includes titration) | **$4 618** | NA | 2021 | Price per pack (pre-filled pens of 6 mg/mL) = $382.22  Pens per pack = 5 pens of 3 mL each  Pack content = 90 mg  Cost per mg = $4.2469  Cost of needles per annum = $39.05/100 needles per pack (116) × 365.25 needles/year × ~100% privately reimbursed $143  Treatment initiation : [[33](#_ENREF_33)]   - Week 1 titration period dose: 0.6 mg *7 = 4.2 mg - Week 2 titration period dose: 1.2 mg *7 = 8.4 mg - Week 3 titration period dose: 1.8 mg *7 = 12.6 mg - Week 4 titration period dose: 2.4 mg *7 = 16.8 mg - Maintenance dose per day (3 mg/day)   Cost of liraglutide per dose on maintenance dose = $4.2469 * 3 mg/day = $12.74  Total cost of liraglutide in titration phase = $4.2469 *(4.2 mg + 8.4 mg + 12.6 mg + 16.8 mg) + $143*(28/365) = $189.31  Total cost in 1st year = total cost in 1st quarter ($1 020.67) + total cost per quarter ($1 199.04) * 3 = $4 618 |
| Liraglutide 3.0 mg daily - follow-up year | **$4 796** | NA | 2021 | Total cost of liraglutide maintenance dose per quarter ($1 199.04) [[34](#_ENREF_34)]* 4 = $4 796.16 |
| Orlistat (3× 120 mg daily) | **$1 816** | NA | 2021 | Cost per capsule: $1.657  Cost of maintenance dose per day: $1.657*3 = $4.972  Total cost of orlistat maintenance dose per year: $4.972*365.25 = $1 816.10 [[34](#_ENREF_34), [35](#_ENREF_35)] |
| NB 32 (4× 8 mg / 90 mg tablets daily), 1st year | **$3 143** | NA | 2021 | Cost per tablet = $2.21  Cost of loading phase:   - Week 1: 7* $2.21 = $15.50 - Week 2: 7*2* $2.21 = $31.01 - Week 3: 7*3* $2.21 = $46.51   Cost of maintenance dose per week: 7*4*$2.21 = $62.02  Total cost of NB-32 maintenance dose, per year = $15.50 + $31.01 + $46.51 + $62.02 * (52.18-3) = $3 143 [[34](#_ENREF_34), [36](#_ENREF_36)] |
| NB 32 (4× 8 mg / 90 mg tablets daily), subsequent years | **$3 236** | NA | 2021 | Total cost of NB-32 maintenance dose, per year: $62.02 * 52.18 = $3 236 [[34](#_ENREF_34), [36](#_ENREF_36)] [[36](#_ENREF_36), [37](#_ENREF_37)] |
| **Monitoring costs for obesity, annual** | | | | |
| Direct healthcare and non-healthcare costs to private payers | **$0** | $0 | NA | Not covered, thus assumed zero |
| Direct healthcare costs to public payers | **$210** | $210 | 2021 | Assumed 4 GP visits per year and lab tests 2 times a year that include HbA_1c_, lipid panel, ALT [[9](#_ENREF_9), [38](#_ENREF_38)]  Calculation:  4 visits*$45.9 [[39](#_ENREF_39)](A006 Repeat consultation)= $184  2 × ($7.25 + $1.28 + $2 + $1.28 + $1.28) [[40](#_ENREF_40)] = $26  Total: $184 + $26 = $210 |
| Indirect costs - work productivity losses | **$0** | $0 | NA | Not considered |
| **Total societal** | **$210** |  |  | Sum of the above |
| **Diet and exercise (addition to obesity monitoring costs above)** | | | | |
| Direct healthcare and non-healthcare costs to private payers | **$4 303** | $4 303 | 2021 | Based on programs recommended by Canada Obesity Management Clinical guideline Wharton et al [[38](#_ENREF_38)]  Average annual cost of 4 programs recommended by the guideline:   - Weight Watcher: 6.95 * 4 * 12 = $333.6 [[41](#_ENREF_41)] - OPTIFAST: $75 * 15 [[42](#_ENREF_42)] - Nutrisystem: $10.9 a day * 7 days * 52.18 weeks = $3 981 [[43](#_ENREF_43)]   Jenny Craig: $24 a day * 7 days * 52.18 weeks: $8 766 [[44](#_ENREF_44)] |
| Direct healthcare costs to public payers | **$0** | $0 | NA | No national-level program reimbursed under the public payer, thus cost assumed zero |
| Indirect costs (work productivity losses) | **$0** | $0 | NA | Not considered |
| **Total societal** | **$ 4 303** |  |  | Sum of the above |
| **Blood pressure treatment** | | | | |
| Direct healthcare and non-healthcare costs to private payers | **$0** | $0 | NA | Included in public healthcare costs |
| Direct healthcare costs to public payers | **$113** | $113 | 2021 | Ramipril (strength: 5 mg; dose: 5 mg/day) IQVIA Pharma Stat and Delta PA [[34](#_ENREF_34)]  Calculation:  Price per tablet ($0.31) *365.25 days = $113.23 |
| Indirect costs (work productivity losses) | **$0** | $0 | NA | Not considered |
| **Total societal** | **$113** |  |  | Sum of the above |
| **Treatment-related adverse events** | | | | |
| *Severe GI event* | | | | |
| Direct healthcare and non-healthcare costs to private payers | **$45** | $45 | 2021 | Cost of medically necessary ambulance [[45](#_ENREF_45)] |
| Direct healthcare costs to public payers | **$365** | $365 | 2021 | CADTH CDR Signifor (2015) [[46](#_ENREF_46)] |
| Indirect costs (work productivity losses) | **$0** | $0 | NA | Not considered |
| **Total societal** | **$410** |  |  |  |
| *Severe hypoglycemia* | | | | |
| Direct healthcare and non-healthcare costs to private payers | **$45** | $45 | 2021 | Cost of medically necessary ambulance [[45](#_ENREF_45)] |
| Direct healthcare costs to public payers | **$2 352** | $2 352 | 2021 | CADTH Optimal Use report (2013) [[47](#_ENREF_47)] |
| Indirect costs (work productivity losses) | **$0** | $0 | NA | Not considered |
| **Total societal** | **$2 397** |  |  |  |
| *Non-severe hypoglycemia* | | | | |
| Direct healthcare and non-healthcare costs to private payers | **$7** | $7 | 2021 | Aronson R,et al (2018) [[48](#_ENREF_48)]; CADTH Therapeutic revew (2017) [[49](#_ENREF_49)]; Pollock RF et al (2019) |
| Direct healthcare costs to public payers | **$7** | $7 | 2021 | Aronson R,et al (2018) [[48](#_ENREF_48)]; CADTH Therapeutic revew (2017) [[49](#_ENREF_49)]; Pollock RF et al (2019) |
| Indirect costs (work productivity losses) | **$0** | $0 | NA | Not considered |
| **Total societal** | **$14** |  |  |  |
| **Applied to health states - exclude acute care costs related to events** | | | | |
| *T2D microvascular complications costs (including T2D pharmacy costs above)* | | | | |
| Direct healthcare costs to private payers | **$2 929** | $2 497 | 2021 | Provincial average (Ontario) out-of-pocket expenses across all income categories, all inflated to 2021  (2 073.5+2 594.7+2 821.45)/3 = $2 497 [[50](#_ENREF_50)] |
| Direct healthcare and non-healthcare costs to public payers | **$2 352** | $2 352 | 2021 | Comparative cost analysis of adult obesity in Alberta, prepared by Alberta RWE Consortium, S Klarenbach et al, Novo Nordisk data on file [[44](#_ENREF_44)] |
| Indirect costs (work productivity losses) | **$639** | $532 | 2010 | Cost is for absent workdays and productivity losses in a 3-month period ($133.08) multiplied by 4 [[69](#_ENREF_69)] |
| **Total societal** | **$5 920** |  |  | Sum of the above |
| *Prediabetes* | | | | |
| Direct healthcare costs to private payers | **$0** | $0 | NA | The costs are assumed to be included in obesity monitoring costs |
| Direct healthcare and non-healthcare costs to public payers | **$106** | $106 | 2021 | Comparative cost analysis of adult obesity in Alberta, prepared by Alberta RWE Consortium, S Klarenbach et al, Novo Nordisk data on file [[51](#_ENREF_51)] |
| Indirect costs (work productivity losses) | **$0** | $0 | NA | Assumed zero |
| **Total societal** | **$106** |  |  | Sum of the above |
| *Cancer treatment colon in 1st year* | | | | |
| Direct healthcare and non-healthcare costs to private payers | **$7 954** | $7 751 | 2020 | The costs included prescription drugs, in-home healthcare, homemaking services, complementary and alternative medicine, vitamins and supplements, family care, other health professionals, travel/parking, accommodations/meals, devices/equipment, and ‘other’ costs [[52](#_ENREF_52)]  Calculation:  Healthcare costs ($574*365.25/28) + travel ($179) + Parking ($84) = $7,751 |
| Direct healthcare costs to public payers (colorectal) | **$30 646** | $24 952 | 2009 | Accounting for a half-cycle correction, costs for the first 6 months after diagnosis were used for colorectal cancer and a mean of males and females was calculated [[53](#_ENREF_53)]  Calculation:  ($25 138 in males + $24 765 in females)/2 = $24 952 |
| Indirect costs (work productivity losses) | **$15 278** | $14 888 | 2020 | Indirect cost includes caregiver time ($9 145) and lost earnings per year ($5 743) [[54](#_ENREF_54)] |
| **Total societal** | **$53 878** |  |  | Sum of the above |
| *Cancer treatment breast in 1st year* | | | | |
| Direct healthcare and non-healthcare costs to private payers | **$7 954** | $7 751 | 2020 | Calculated as described above [[52](#_ENREF_52)] |
| Direct healthcare costs to public payers | **$15 008** | $12 219 | 2009 | Accounting for a half-cycle correction, costs for the first 6 months after diagnosis were used for breast cancer [[53](#_ENREF_53)] |
| Indirect costs (work productivity losses) | **$15 278** | $14 888 | 2020 | Indirect cost includes caregiver time ($9 145) and lost earnings per year ($5 743) [[54](#_ENREF_54)] |
| **Total societal** | **$38 240** |  |  | Sum of the above |
| *Cancer treatment endometrial in 1st year* | | | | |
| Direct healthcare and non-healthcare costs to private payers | **$7 954** | $7 751 | 2020 | Calculated as described above [[52](#_ENREF_52)] |
| Direct healthcare costs to public payers | **$14 841** | $12 083 | 2009 | Accounting for a half-cycle correction, costs for the first 6 months after diagnosis were used for endometrial cancer [[53](#_ENREF_53)] |
| Indirect costs (work productivity losses) | **$15 278** | $14 888 | 2020 | Indirect cost includes caregiver time ($9 145) and lost earnings per year ($5 743) [[54](#_ENREF_54)] |
| **Total societal** | **$38 073** |  |  | Sum of the above |
| *Cancer treatment (average of colon, breast, endometrial) in follow-up year* | | | | |
| Direct healthcare and non-healthcare costs to private payers | **$5 678** | $5 533 | 2020 | As per reported costs beyond the 1st year [[52](#_ENREF_52)]  Calculation:  Healthcare costs ($404*365.25/28) + travel ($179) + parking ($84) = $5 533 |
| Direct healthcare costs to public payers | **$6 329** | $5 153 | 2009 | The mean annual costs of 3 cancer types (colorectal, breast, endometrial) at continuing phase were used to calculate the average annual costs in follow-up years [[53](#_ENREF_53)]  Calculation:  Colorectal: ($5 446 in males + $5 349 in females)/2  Breast: $6 741  Endometrial: $3 320  Cost input: ($5 446 + $5 349)/2 + $6 741 + $3 320)/3 = $5 153 |
| Indirect costs (work productivity losses) | **$10 759** | $10 485 | 2020 | A ratio of 1st-year to follow-up year costs (CAD574/CAD404 = 1.42) based on Longo et al [[52](#_ENREF_52)] was applied to the average of the 3 cancer treatment costs in the above ($14 888) to estimate the costs in the follow-up year  Calculation:  $14 888/1.42 = $10 485 |
| **Total societal** | **$22 766** |  |  | Sum of the above |
| *MI 1st year, excluding acute event cost* | | | | |
| Direct healthcare and non-healthcare costs to private payers | **$1 434** | $1 152 | 2008 | The costs include medication in the 1st year [[55](#_ENREF_55)]. First-year costs are cycle corrected by division with 2, assuming event occurs halfway through the year: $2,305 inflated to $2,868 and divided by 2 for cycle correction |
| Direct healthcare costs to public payers | **$2 970** | $2 387 | 2008 | The first-year follow-up costs were used that included post-event hospitalizations ($3 966), physician fees ($366) and community care ($1 325).  First-year costs are cycle corrected by division with 2, assuming event occurs halfway through the year [[56](#_ENREF_56)]  Calculation:  ($3 966 + $366 + $1 325)/2 = $2 387 |
| Indirect costs (work productivity losses) | **$5 900** | $4 578 | 2006 | First-year costs are cycle corrected by division with 2, assuming event occurs halfway through the year:  $9 156 /2 = $4 578 [[57](#_ENREF_57)] |
| **Total societal** | **$10 304** |  |  | Sum of the above |
| *Unstable angina 1st year, excluding acute event cost* | | | | |
| Direct healthcare and non-healthcare costs to private payers | **$2 185** | $1 634 | 2004 | Reported direct costs that included out-of-pocket expenses [[58](#_ENREF_58)]. First-year costs are cycle corrected by division with 2, assuming event occurs halfway through the year |
| Direct healthcare costs to public payers | **$1 932** | $1 760 | 2016 | First-year costs of $3 267 are inflated to 2021 $4 370 and divided by two for cycle correction, assuming event occurs halfway through the year [[59](#_ENREF_59)] |
| Indirect costs (work productivity losses) | **$8 671** | $6 482 | 2004 | Value represents indirect time from caregiving (including leisure and work) and lost earnings. First-year costs are cycle corrected by division with 2, assuming event occurs halfway through the year:  $12 963 /2 = $ 6 482 [[58](#_ENREF_58)] |
| **Total societal** | **$12 788** |  |  | Sum of the above |
| *Post-ACS (MI or angina, in year following the event)* | | | | |
| Direct healthcare and non-healthcare costs to private payers | **$1 598** | $1 284 | 2008 | Costs included medication expenses in subsequent years [[55](#_ENREF_55)] |
| Direct healthcare costs to public payers | **$1 500** | $1 325 | 2014 | Reported 3-year costs of post-MI follow-up were annualized [[56](#_ENREF_56)]  Calculation:  $3 974.14/3 = $1 325 |
| Indirect costs (work productivity losses) | **$1 009** | $783 | 2006 | Reported costs for years 2–5 after the index events where productivity costs for year 5 were equal $0, assuming the patients return to the ‘at-risk’ state, but no further productivity costs are incurred. The costs for years 2–4 were spread across the 20-year time horizon and annualized [[57](#_ENREF_57)]  Calculation:  Average of post-MI annual costs: ($5 966 + $6 254 + $6 281) and post-revascularization annual costs for 3 years after the event ($5 002 + $ 4 055 + $ 3 749) = $15 654  Annualized across a 20-year time horizon: $15 654/20 = $783 |
| **Total societal** | **$4 107** |  |  | Sum of the above |
| *Stroke 1st year, excluding acute event cost* | | | | |
| Direct healthcare and non-healthcare costs to private payers | **$1 369** | $1 024 | 2004 | The costs included medication costs ($1 624), assistive devices or home renovations ($423)[[60](#_ENREF_60)]. First-year costs are cycle corrected by division with 2, assuming event occurs halfway through the year  Calculation:  ($1,624 + $423)/2 = $1,024 |
| Direct healthcare costs to public payers | **$11 869** | $11 230 | 2018 | Proportion of males (51%, $60 000) and females (49%, $54 753) in the sample were used to calculate a weighted average total cost reported by Yu et al [[61](#_ENREF_61)]. Of note, inpatient care costs reported by Yu et al include instances of subsequent inpatient care within 1 year following the index stroke event.  First-year costs are cycle corrected by division with 2, assuming event occurs halfway through the year  Next, acute care costs ($17 142 inflated to values with a factor of 1.02 to 2018) obtained from OCCI were subtracted from 1-year total cost estimates reported in Yu et al [[61](#_ENREF_61)]  Calculation:  ($60 000*0.51 + $54 753*0.49)/2 –$17 142*1.02 = $11 230 |
| Indirect costs (work productivity losses) | **$5 323** | $5 149 | 2019 | Productivity losses in stroke survivors were estimated at 53 days a year amounting to $10 298 [[62](#_ENREF_62)]  First-year costs are cycle corrected by division with 2, assuming event occurs halfway through the year |
| **Total societal** | **$18 561** |  |  | Sum of the above |
| *Transient ischemic attack, 1st year, excluding acute event cost* | | | | |
| Direct healthcare and non-healthcare costs to private payers | **$1 129** | $844 | 2004 | The costs included medication costs, assistive devices or home renovations. First-year costs are cycle corrected by division with 2, assuming event occurs halfway through the year [[60](#_ENREF_60)]  Calculation:  ($1 574 + $114)/2 = $844 |
| Direct healthcare costs to public payers | **$13 892** | $12 089 | 2013 | Reported monthly costs were multiplied by 11 months. First-year costs are cycle corrected by division with 2, assuming event occurs halfway through the year [[63](#_ENREF_63)]  Calculation:  ($2 198 (per month) * 11 months)/2 = $12 089 |
| Indirect costs (work productivity losses) | **$181** | $136 | 2004 | First-year costs are cycle corrected by division with 2, assuming event occurs halfway through the year [[60](#_ENREF_60)] |
| **Total societal** | **$15 202** |  |  | Sum of the above |
| *Post-stroke (stroke and TIA, in year following the event)* | | | | |
| Direct healthcare and non-healthcare costs to private payers | **$1 012** | $756 | 2004 | Assume perpetual medication and device post-stroke costs using last 5 months of 1st-year costs, and post-TIA costs from 1st-year costs [[60](#_ENREF_60)]. Weights for ischemic vs. hemorrhagic stroke were sourced from Yu et al [[61](#_ENREF_61)]  Ischemic stroke cost, annual: 1 624+423 = 2 047; hemorrhagic stroke cost, annual: 660+177=837  Weighted average cost stroke for 5 months: (2,047*87%+837 *13%)/12*5 = 787.4  TIA cost: ($1 574 + $114)/12 * 5 = $703  Weighted average cost post-stroke and TIA (135/365 * $703) + (230/365 * $787.4) = $756, whereby 135/365 represent the proportion of TIA and 230/365 strokes in the total. |
| Direct healthcare costs to public payers | **$3 889** | $5 097 | 2005 | Weighted average cost of TIA and stroke from year 1 from event. Inputs from Goeree et al (2005) [[60](#_ENREF_60)] were used for weights and costs; the proportions of ischemic vs. hemorrhagic stroke were sourced from Yu (2021) [[61](#_ENREF_61)].  Assuming the patients receive long-term care/home care, prescription drugs, assistive devices, and outpatient visits [[60](#_ENREF_60)]  Calculation:  Post-TIA costs: $207 + $1 574 + $483 + $114 = $2 378  Post-stroke costs (ischemic): $2 325 + $1 624 + $607 + $423 = $4 979  Post-stroke costs (hemorrhagic): $2 124 + $660 + $460 + $177 = $3 421  Post-stroke costs (weighted): $4 979 * 0.87 + $3 421 * 0.13 = $4 777  Weights:  TIA – 135/265 = 0.37  Stroke – 230/265 = 0.63  Post-TIA/stroke total annual cost= $2 378*0.37 + $4.777*0.63 = $3 889 |
| Indirect costs (work productivity losses) | **$2 325** | $1 893 | 2009 | The approach to estimate the cost assumes a 4-year recovery period after which productivity costs are 0; the total of 4-year costs are spread across the 20-year time horizon  The annual costs were calculated based on the costs incurred in the 7–12 month post-index period ($22 373, of which 42.3% were indirect costs) [[57](#_ENREF_57), [64](#_ENREF_64)]  Calculation:  ($22 373 * 0.423 * 4)/20 = $1 893 |
| **Total societal** | **$7 226** |  |  | Sum of the above |
| *Sleep apnea cost* | | | | |
| Direct healthcare and non-healthcare costs to private payers | **$1 023** | $949 | 2017 | CPAP costs for Ontario (private portion, 2017): assuming 5-year amortization: ($1 200 − $645)/5 = $111  Annual cost of refills included 1 each of new mask/headgear, tubing, filter ($822*1.02+111 = $838) [[65](#_ENREF_65), [66](#_ENREF_66)] |
| Direct healthcare costs to public payers | **$176** | $129  (CPAP only) | 2017 | Assumed an annual follow-up visit to a specialist (A947) [[39](#_ENREF_39)]  CPAP for Ontario (public portion: $645; 2017): assuming 5-year amortization [[66](#_ENREF_66), [67](#_ENREF_67)]  Calculation:  CPAP costs ($645/5 = $129; 2017) + a physician visit ($36.85; 2021) = $176. note that the CPAP costs only were inflated |
| Indirect costs (work productivity losses) | **$0** | $0 | NA | Not available, assumed zero |
| **Total societal** | **$1 199** |  |  | Sum of the above |
| **Applied to events** | | | | |
| *MI, fatal and non-fatal events* | | | | |
| Direct healthcare and non-healthcare costs to private payers | **$45** | $45 | 2021 | Assume patient transported to hospital via medically necessary ambulance [[45](#_ENREF_45)] |
| Direct healthcare costs to public payers | **$9 261** | $8 591 | 2017 | Average acute inpatient care for the following ICD10CA codes sourced from OCCI: I210,I211,I212,I213,I214,I219 [[68](#_ENREF_68)] |
| Indirect costs (work productivity losses) | **$0** | $0 | NA | Included in state cost |
| **Total societal** | **$9 306** |  |  | Sum of the above |
| *Unstable angina, fatal and non-fatal events* | | | | |
| Direct healthcare and non-healthcare costs to private payers | **$45** | $45 | 2021 | Assume patient transported to hospital via medically necessary ambulance [[45](#_ENREF_45)] |
| Direct healthcare costs to public payers | **$6 033** | $5 597 | 2017 | Average acute inpatient care for ICD10CA codes sourced from OCCI: I200 [[68](#_ENREF_68)] |
| Indirect costs (work productivity losses) | **$0** | $0 | NA | Included in state cost |
| **Total societal** | **$6 078** |  |  | Sum of the above |
| *Stroke, fatal and non-fatal events* | | | | |
| Direct healthcare and non-healthcare costs to private payers | **$45** | $45 | 2021 | Assume patient transported to hospital via medically necessary ambulance [[45](#_ENREF_45)] |
| Direct healthcare costs to public payers | **$18 479** | $17 142 | 2017 | Average acute inpatient care for ICD10CA codes sourced from OCCI: I60, I61, I63, I64 [[68](#_ENREF_68)] |
| Indirect costs (work productivity losses) | **$0** | $0 | NA | Included in state cost |
| **Total societal** | **$18 524** |  |  | Sum of the above |
| *TIA event, non-fatal* | | | | |
| Direct healthcare and non-healthcare costs to private payers | **$45** | $45 | 2021 | Assume patient transported to hospital via medically necessary ambulance [[45](#_ENREF_45)] |
| Direct healthcare costs to public payers | **$4 503** | $4 177 | 2017 | Average acute inpatient care for ICD10CA codes sourced from OCCI: G459 [[68](#_ENREF_68)] |
| Indirect costs (work productivity losses) | **$0** | $0 | NA | Included in state cost |
| **Total societal** | **$4 548** |  |  | Sum of the above |
| *Bariatric surgery, fatal and non-fatal* | | | | |
| Direct healthcare and non-healthcare costs to private payers | **$0** | $0 | NA | Covered by public payers only |
| Direct healthcare costs to public payers, pre-operative management | **$1 797** | $1 667 | 2017 | Costs incurred in the 3 months prior to the procedure [[69](#_ENREF_69)] |
| Direct healthcare costs to public payers, gastric bypass procedure | **$20 804** | $17 883 | 2012 | Procedure cost [[70](#_ENREF_70)] |
| Direct healthcare costs to public payers, laparoscopic banding procedure | **$12 181** | $10 471 | 2012 | Procedure cost [[70](#_ENREF_70)] |
| Direct healthcare costs to public payers, sleeve gastrectomy procedure | **$13 884** | $11 934 | 2012 | Procedure cost [[70](#_ENREF_70)] |
| Direct healthcare costs to public payers, post-operative follow-up | **$11 676** | $10 831 | 2017 | Mean net cost of an RYGB for total health care expenditures incurred over the 5-year period [[69](#_ENREF_69)]. |
| Direct healthcare costs to public payers, complications (leaks) | **$0** | $0 | NA | Assumed included in post-operative follow-up [[69](#_ENREF_69)] |
| Indirect costs, work productivity losses | **$15 044** | $13 700 | 2016 | Includes the costs of income transfer payments (i.e., income for disabled persons, disability and employment insurance benefits), as well as the cost of productivity loss, as measured by the friction method [[71](#_ENREF_71)] |
| **Total societal, non-fatal events** | **$45 745** | $0 | NA | Sum of pre- post- operative and weighted average of procedure costs plus indirect costs  The weighted [[72](#_ENREF_72)] average of the costs of 3 main types of bariatric surgeries [[69](#_ENREF_69)] using the following percentages:  Gastric bypass – 53%  Laparoscopic banding – 19%  Sleeve gastrectomy – 28% |
| **Total societal, fatal events** | **$19 025** |  |  | Sum of pre- operative and weighted average of procedure costs, excludes indirect costs and post-operative costs |
| *Knee replacement, fatal and non-fatal* | | | | |
| Direct healthcare and non-healthcare costs to private payers | **$8 565** | $7 362 | 2021 | The indirect costs include a variety of components in the study, including drugs costs, alternative healthcare providers, travel, time off work, etc. [[73](#_ENREF_73)] |
| Direct healthcare costs to public payers: non-fatal events only | **$10 775** | $10 500 | 2020 | The amount includes inpatient procedure, no rehabilitation costs [[73](#_ENREF_73), [74](#_ENREF_74)] |
| Indirect costs (work productivity losses) | **$0** | $0 | NA | No data found, assumed zero |
| **Total societal** | **$19 340** |  |  | Sum of the above |

*ACE* angiotensin-converting enzyme, *ACS* acute coronary syndrome, *ALT* alanine aminotransferase, *CAD* Canadian dollar, *CPAP* continuous positive airway pressure, *GI* gastrointestinal, *GP* general physician, *HbA_1c_* glycated hemoglobin, *ICD* International Classification of Diseases, *MI* myocardial infarction; *NA* not applicable, *OCCI* Ontario Care Costing Initiative, *OOP* out-of-pocket, *RYGB* Roux-en-Y gastric bypass, *RWE* real world evidence, *TIA* transient ischemic attack, *T2D* type 2 diabetes.

Supplementary Table 12. Model used for prediction of body mass index-dependent utilities.

| Variable | Coefficient applied in model | |
| --- | --- | --- |
|  | **Females** | **Males** |
| Model intercept | 1.124393 | 0.794542 |
| Age (years) | −0.000955 | −0.000203 |
| Heart or circulatory diseases (excluding hypertension) | −0.061420 | −0.084805 |
| Hypertension | −0.006639 | −0.012301 |
| Smoking status |  |  |
| Current smoker | −0.017698 | 0.021579 |
| Previous smoker | −0.015345 | −0.002349 |
| Never smoked (reference, thus coefficient = 0) | 0.000000 | 0.000000 |
| Body mass index |  |  |
| Linear effect | −0.006460 | 0.017277 |
| Quadratic effect | 0.000078 | −0.000514 |
| Cubic effect | −0.000001 | 0.000004 |
| Prediabetes status at baseline | −0.003935 | −0.002441 |

Supplementary Table 13. Disutilities associated with health states and acute events.

|  | Mean | SEM |
| --- | --- | --- |
| Disutility applied in health state | | |
| Type 2 diabetes [[75](#_ENREF_75)] | −0.029 | 0.007 |
| Post-acute coronary syndrome [[76](#_ENREF_76)] | −0.037 | 0.009 |
| Obstructive sleep apnea* | 0 | 0 |
| Cancer [[75](#_ENREF_75)] | −0.073 | 0.018 |
| Post-stroke [[76](#_ENREF_76)] | −0.035 | 0.009 |
| Post-diabetes | 0.000 | 0.000 |
| Disutility per event | | |
| Bariatric surgery [[77](#_ENREF_77)] | −0.184 | 0.046 |
| Acute coronary syndromes [[78](#_ENREF_78)] | −0.129 | 0.032 |
| Musculoskeletal [[76](#_ENREF_76)] | −0.023 | 0.006 |
| Stroke [[78](#_ENREF_78)] | −0.181 | 0.045 |
| Transient ischemic attacks [[76](#_ENREF_76)] | −0.033 | 0.008 |
| Severe gastrointestinal events [[79](#_ENREF_79)] | -0.05/52 = −0.001 | 0.0002 |
| Severe hypoglycemia [[80](#_ENREF_80)] | −0.015 | 0.004 |
| Non-severe hypoglycemia [[80](#_ENREF_80)] | −0.0062 | 0.002 |

*Included in baseline utility. *SEM* standard error of the mean.

Supplementary Table 14. Upper and lower values tested in univariate sensitivity analyses on model parameters.

| # |  | Base value | High variation | Low variation |
| --- | --- | --- | --- | --- |
| 1 | Baseline characteristics from | STEP trials | Wharton et al  (Canada) | Ghusn et al (US) |
| 2 | Menopause age | 49.80 | 50.00 | 49.70 |
| 3 | Prop. Histoty of ACS/stroke at baseline | 0.04 | 0.06 | 0.03 |
| 4 | Natural weight increase per year (Kg) | 0.46 | 0.57 | 0.34 |
| 5 | Maximum age for weight increase | 68.00 | 85.00 | 51.00 |
| 6 | Weight loss vs. baseline 4-9 mths, semaglutide 2.4 mg | -0.12 | -0.12 | -0.13 |
| 7 | Weight loss vs. baseline 10-12 mths, semaglutide 2.4 mg | -0.16 | -0.15 | -0.17 |
| 8 | Weight loss vs. baseline 4-9 mths, semaglutide 2.4 mg | -0.03 | -0.02 | -0.03 |
| 9 | Weight loss vs. baseline 10-12 mths, diet & exercise | -0.03 | -0.02 | -0.04 |
| 10 | Weight loss vs. baseline 4-9 mths, diet & exercise | -0.08 | -0.05 | -0.11 |
| 11 | Weight loss vs. baseline 10-12 mths, liraglutide 3.0 mg | -0.12 | -0.09 | -0.15 |
| 12 | Weight loss vs. baseline 4-9 mths, orlistat | -4% | -1% | -7% |
| 13 | Weight loss vs. baseline 10-12 mths, orlistat | -7% | -4% | -11% |
| 14 | Weight loss vs. baseline 4-9 mths, NB-32 | -0.06 | -0.03 | -0.10 |
| 15 | Weight loss vs. baseline 10-12 mths, NB-32 | -0.10 | -0.06 | -0.13 |
| 16 | Weight-loss maintenance per year, pharmacotherapy | 0.91 | 1.00 | 0.77 |
| 17 | Weight-loss maintenance per year, diet & exercise | 0.70 | 1.00 | 0.10 |
| 18 | SBP change vs. baseline 4-9 mths, semaglutide 2.4 mg | -6.24 | -5.15 | -7.34 |
| 19 | SBP change vs. baseline 10-12 mths, semaglutide 2.4 mg | -6.56 | -7.75 | -5.38 |
| 20 | SBP change vs. baseline 4-9 mths, liraglutide 3.0 mg | -4.48 | -2.22 | -6.74 |
| 21 | SBP change vs. baseline 10-12 mths, liraglutide 3.0 mg | -4.80 | -7.06 | -2.54 |
| 22 | SBP change vs. baseline 4-9 mths, diet and exercise | -0.24 | 1.03 | -1.51 |
| 23 | SBP change vs. baseline 10-12 mths, diet and exercise | -0.76 | -2.08 | 0.55 |
| 24 | SBP change vs. baseline 4-9 mths, orlistat | -5.77 | -4.90 | -6.64 |
| 25 | SBP change vs. baseline 10-12 mths, orlistat | -6.09 | -6.96 | -5.22 |
| 26 | SBP change vs. baseline 4-9 mths, NB-32 | -0.27 | 3.96 | -4.50 |
| 27 | SBP change vs. baseline 10-12 mths, NB-32 | -0.59 | -4.82 | 3.64 |
| 28 | Total chol. change vs. baseline 4-9 mths, semaglutide 2.4 mg | -15.27 | -14.90 | -15.63 |
| 29 | Total chol. change vs. baseline 10-12 mths, semaglutide 2.4 mg | -6.76 | -6.61 | -6.91 |
| 30 | Total chol change vs. baseline 4-9 mths, diet and exercise | 1.09 | 1.13 | 1.06 |
| 31 | Total chol. change vs. baseline 10-12 mths, diet and exercise | 0.80 | 0.84 | 0.75 |
| 32 | Total chol. change vs. baseline 4-9 mths, liraglutide 3.0 mg | -13.95 | -9.80 | -18.10 |
| 33 | Total chol. change vs. baseline 10-12 mths, liraglutide 3.0 mg | -5.43 | -1.06 | -9.80 |
| 34 | Total chol. change vs. baseline 4-9 mths, orlistat | -15.27 | -15.27 | -15.27 |
| 35 | Total chol. change vs. baseline 10-12 mths, orlistat | -6.76 | -6.76 | -6.76 |
| 36 | Total chol. change vs. baseline 4-9 mths, NB-32 | -15.27 | -15.27 | -15.27 |
| 37 | Total chol. change vs. baseline 10-12 mths, NB-32 | -6.76 | -6.76 | -6.76 |
| 38 | HDL chol. change vs. baseline 4-9 mths, semaglutide 2.4 mg | -3.52 | -3.44 | -3.60 |
| 39 | HDL chol. change vs. baseline 10-12 mths, semaglutide 2.4 mg | 3.44 | 3.52 | 3.35 |
| 40 | HDL chol change vs. baseline 4-9 mths, diet and exercise | -0.62 | -0.60 | -0.64 |
| 41 | HDL chol. change vs. baseline 10-12 mths, diet and exercise | 1.08 | 1.11 | 1.05 |
| 42 | HDL change vs. baseline 4-9 mths, liraglutide 3.0 mg | -3.97 | -2.72 | -5.22 |
| 43 | HDL change vs. baseline 10-12 mths, liraglutide 3.0 mg | 2.91 | 4.35 | 1.48 |
| 44 | HDL chol. change vs. baseline 4-9 mths, orlistat | -3.52 | -3.52 | -3.52 |
| 45 | HDL chol. change vs. baseline 10-12 mths, orlistat | 3.44 | 3.44 | 3.44 |
| 46 | HDL chol. change vs. baseline 4-9 mths, NB-32 | -3.52 | -3.52 | -3.52 |
| 47 | HDL chol. change vs. baseline 10-12 mths, NB-32 | 3.44 | 3.44 | 3.44 |
| 48 | T2D HbA1c change vs. baseline 4-9 mths, semaglutide 2.4 mg | -1.8% | -1.8% | -1.9% |
| 49 | T2D HbA1c change vs. baseline 10-12 mths, semaglutide 2.4 mg | -1.9% | -1.8% | -2.0% |
| 50 | T2D HbA1c change vs. baseline 4-9 mths, diet and exercise | -0.3% | -0.2% | -0.4% |
| 51 | T2D HbA1c change vs. baseline 10-12 mths, diet and exercise | -0.3% | -0.2% | -0.4% |
| 52 | T2D HbA1c change vs. baseline 4-9 mths, liraglutide 3.0 mg | -1.1% | 0.6% | -2.8% |
| 53 | T2D HbA1c change vs. baseline 10-12 mths, liraglutide 3.0 mg | -1.2% | 0.5% | -2.9% |
| 54 | T2D HbA1c hange vs. baseline 4-9 mths, orlistat | -1.0% | 0.4% | -2.4% |
| 55 | T2D HbA1c change vs. baseline 10-12 mths, orlistat | -1.1% | 0.3% | -2.5% |
| 56 | T2D HbA1c change vs. baseline 4-9 mths, NB-32 | -1.1% | 0.6% | -2.8% |
| 57 | T2D HbA1c change vs. baseline 10-12 mths, NB-32 | -1.2% | 0.5% | -2.9% |
| 58 | Prob. prediabetes reversal, semaglutide 2.4 mg | 0.84 | 0.87 | 0.81 |
| 59 | Prob. prediabetes reversal, diet & exercise | 0.48 | 0.54 | 0.42 |
| 60 | Prob. prediabetes reversal, liraglutide 3.0 mg | 0.86 | 0.90 | 0.81 |
| 61 | Prob. prediabetes reversal, NB-32 | 0.80 | 0.93 | 0.68 |
| 62 | Prob. prediabetes reversal, orlistat | 0.80 | 0.93 | 0.68 |
| 63 | Prob. loss of drug-induced normoglycemia, pharmacotherapy | 0.08 | 0.26 | 0.00 |
| 64 | Prob. loss of drug-induced normoglycemia, diet & exercise | 0.39 | 0.81 | 0.00 |
| 65 | Prob. non-response, semaglutide 2.4 mg | 0.21 | 0.25 | 0.18 |
| 66 | Prob. non-response, diet and exercise | 0.76 | 0.81 | 0.71 |
| 67 | Prob. non-response, liraglutide 3.0 mg | 0.32 | 0.42 | 0.22 |
| 68 | Prob. non-response, NB-32 | 0.53 | 0.78 | 0.27 |
| 69 | Prob. non-response, orlistat | 0.54 | 0.68 | 0.39 |
| 70 | Treatment duration (years) | 2.00 | 10.00 | 5.00 |
| 71 | Prob. CVD_is_MI | 0.35 | 0.35 | 0.35 |
| 72 | Prob MI is fatal | 0.31 | 0.47 | 0.17 |
| 73 | Prob. CVD_is_angina | 0.39 | 0.39 | 0.39 |
| 74 | Prob Angina is fatal | 0.31 | 0.47 | 0.17 |
| 75 | Prob. CVD_is_stroke | 0.26 | 0.26 | 0.26 |
| 76 | Prob stroke is fatal | 0.22 | 0.34 | 0.12 |
| 77 | Prob CVD is TIA | 0.22 | 0.34 | 0.12 |
| 78 | Prob CVD is TIA | 0.22 | 0.34 | 0.12 |
| 79 | Prob KRS is fatal | 0.00 | 0.00 | 0.00 |
| 80 | Prob colon cancer is fatal | 0.30 | 0.46 | 0.17 |
| 81 | Prob breast cancer is fatal | 0.04 | 0.06 | 0.02 |
| 82 | Prob endometrial cancer is fatal | 0.11 | 0.16 | 0.06 |
| 83 | Prob. Cancer mortality, 2+ years | 0.04 | 0.07 | 0.02 |
| 84 | Risk of colon cancer in reference group, males | 0.09% | 0.14% | 0.05% |
| 85 | Risk of colon cancer in reference group, females | 0.07% | 0.10% | 0.04% |
| 86 | Risk of breast cancer in reference group | 0.20% | 0.32% | 0.12% |
| 87 | Risk of endometrial cancer in reference group | 0.05% | 0.08% | 0.03% |
| 88 | Risk of TKR in reference group, under 64 years | 0.00 | 0.00 | 0.00 |
| 89 | Risk of TKR in reference group, over 64 years | 0.00 | 0.00 | 0.00 |
| 90 | Relative risk of death after ACS | 1.30 | 0.00 | 0.00 |
| 91 | Relative risk of death after stroke | 2.00 | 0.00 | 0.00 |
| 92 | Cost Monitoring Pharmacotherapy Public | 210 | 262 | 157 |
| 93 | Cost Monitoring Pharmacotherapy Private | 4 303 | 5 378 | 3 227 |
| 94 | Cost blood pressure treatment Public | 0 | 0 | 0 |
| 95 | Cost blood pressure treatment Private | 113 | 141 | 85 |
| 96 | Cost Severe GI Public | 365 | 456 | 274 |
| 97 | Cost Severe Hypo Public | 2 352 | 2 940 | 1 764 |
| 98 | Cost T2DM Public | 2 352 | 2 940 | 1 764 |
| 99 | Cost T2DM Private | 2 929 | 3 661 | 2 196 |
| 100 | Cost T2DM WPL | 639 | 798 | 479 |
| 101 | Cost PreT2DMPub | 106 | 133 | 80 |
| 102 | Cost ColonCancer_yr1Public | 30 646 | 38 308 | 22 985 |
| 103 | Cost BreastCancer_yr1Public | 15 008 | 18 760 | 11 256 |
| 104 | Cost EndoCancer_yr1Public | 14 841 | 18 551 | 11 130 |
| 105 | Cost Cancer_yr2Pub | 6 329 | 7 911 | 4 747 |
| 106 | Cost ColonCancer_yr1Prod | 15 278 | 19 098 | 11 459 |
| 107 | Cost BreastCancer_yr1Prod | 15 278 | 19 098 | 11 459 |
| 108 | Cost EndoCancer_yr1Prod | 15 278 | 19 098 | 11 459 |
| 109 | Cost Cancer_yr2Prod | 10 759 | 13 449 | 8 069 |
| 110 | Cost MI_yr1Pub | 2 970 | 3 713 | 2 228 |
| 111 | Cost Angina_yr1Pub | 1 932 | 2 415 | 1 449 |
| 112 | Cost Post ACS Pub | 1 500 | 1 875 | 1 125 |
| 113 | Cost Stroke_yr1Pub | 11 869 | 14 837 | 8 902 |
| 114 | Cost TIA_yr1Pub | 13 892 | 17 365 | 10 419 |
| 115 | Cost Post Stroke Pub | 5 097 | 6 371 | 3 822 |
| 116 | Cost MI_yr1Prod | 5 900 | 7 375 | 4 425 |
| 117 | Cost Angina_yr1Prod | 8 671 | 10 838 | 6 503 |
| 118 | Cost Post ACS Prod | 1 009 | 1 261 | 757 |
| 119 | Cost Stroke_yr1Prod | 5 323 | 6 653 | 3 992 |
| 120 | Cost TIA_yr1Prod | 181 | 227 | 136 |
| 121 | Cost Post Stroke Prod | 2 325 | 2 906 | 1 744 |
| 122 | Cost MI_yr1 | 1 434 | 1 793 | 1 076 |
| 123 | Cost Angina_yr1 | 2 185 | 2 732 | 1 639 |
| 124 | Cost Post ACS | 1 598 | 1 998 | 1 199 |
| 125 | Stroke_yr1 | 1 369 | 1 712 | 1 027 |
| 126 | Cost TIA_yr1 | 1 129 | 1 411 | 847 |
| 127 | Cost Post Stroke | 1 012 | 1 265 | 759 |
| 128 | Cost Sleep Apnoea Pub | 176 | 220 | 132 |
| 129 | Cost Sleep Apnoea | 1 023 | 1 279 | 768 |
| 130 | Cost MI nonfatal Pub | 9 261 | 11 576 | 6 946 |
| 131 | Cost MI nonfatal | 45 | 56 | 34 |
| 132 | Cost Angina nonfatal Pub | 6 033 | 7 542 | 4 525 |
| 133 | Cost Angina nonfatal | 45 | 56 | 34 |
| 134 | Cost Stroke nonfatal Pub | 18 479 | 23 098 | 13 859 |
| 135 | Cost Stroke nonfatal | 45 | 56 | 34 |
| 136 | Cost TIA Pub | 4 503 | 5 628 | 3 377 |
| 137 | Cost Knee Replacement nonfatal Pub | 10 775 | 13 469 | 8 081 |
| 138 | Cost Knee Replacement nonfatal | 8 565 | 10 706 | 6 424 |
| 139 | Cost Bariatric Surgery nonfatal Pub | 30 701 | 38 376 | 23 026 |
| 140 | Cost Bariatric Surgery fatal Pub | 19 025 | 23 782 | 14 269 |
| 141 | Cost Bariatric Surgery nonfatal Prod | 15 044 | 18 805 | 11 283 |
| 142 | Discount rate Costs | 0.015 | 0.03 | 0 |
| 143 | Disutility PreT2D | 0.00 | 0.00 | 0.00 |
| 144 | Disutility T2DM | -0.03 | -0.02 | -0.04 |
| 145 | Disutility Post ACS | -0.04 | -0.03 | -0.05 |
| 146 | Disutility Cancer | -0.07 | -0.05 | -0.09 |
| 147 | Disutility Stroke | -0.03 | -0.03 | -0.04 |
| 148 | Disutility Bariatric Surgery | -0.18 | -0.14 | -0.23 |
| 149 | Disutility AC event | -0.13 | -0.10 | -0.16 |
| 150 | Disutility Knee | -0.02 | -0.02 | -0.03 |
| 151 | Disutility Stroke event | -0.18 | -0.14 | -0.23 |
| 152 | Disutility TIA | -0.03 | -0.02 | -0.04 |
| 153 | Discount rate benefits | 0.015 | 0.03 | 0 |
| 154 | Time with osteoarthritis | 3 | 5 | 0 |
| 156 | Catch-up rate from: | STEP 4 | STEP 1 Extension | Ara et al |
| 157 | Baseline utility (BMI EQ-5D) | STEP 1 | Soltoft et al. | Soltoft et al. |
| 158 | Inclusion of obesity pharmacotherapy adverse events | Yes | Yes | No |

*ACS* acute coronary syndrome*, CVD* cardiovascular disease*, GI* gastrointestinal*, HDL* high density lipoprotein*, MI* myocardial infarction*, T2DM* type 2 diabetes mellitus*, TIA* transient ischemic attack*, WPL* work productivity loss

Supplementary Table 15. Breakdown of clinical results

|  |  | Semaglutide 2.4 mg | Liraglutide 3.0 mg | Diet & exercise | Orlistat | NB-32 |
| --- | --- | --- | --- | --- | --- | --- |
| Event rate per 100 patient-years | CV-events | 2.86 | 2.87 | 2.89 | 2.87 | 2.88 |
|  | Knee replacement | 1.22 | 1.22 | 1.23 | 1.23 | 1.23 |
| Patient-years in each health state (undiscounted) | No comorbidity | 6.69 | 6.46 | 5.80 | 6.19 | 6.20 |
|  | OSA | 10.19 | 10.26 | 10.35 | 10.32 | 10.30 |
|  | Pre-T2D | 5.79 | 5.87 | 6.22 | 6.00 | 6.00 |
|  | T2D | 12.22 | 12.32 | 12.62 | 12.45 | 12.44 |
|  | Post ACS | 3.60 | 3.60 | 3.62 | 3.61 | 3.61 |
|  | Cancer | 1.26 | 1.27 | 1.29 | 1.28 | 1.28 |
|  | Post stroke | 1.43 | 1.43 | 1.44 | 1.43 | 1.43 |
| LY & QALYs | Undiscounted LY | 26.35 | 26.30 | 26.21 | 26.25 | 26.26 |
|  | Undiscounted QALYs | 22.46 | 22.39 | 22.28 | 22.34 | 22.34 |
|  | Discounted LY | 21.41 | 21.36 | 21.29 | 21.33 | 21.33 |
|  | Discounted QALYs | 18.32 | 18.27 | 18.18 | 18.22 | 18.23 |
| QALY breakdown by comorbidity (discounted) | No comorbidity | 5.15 | 4.95 | 4.36 | 4.71 | 4.72 |
|  | Pre-T2D | 4.21 | 4.30 | 4.63 | 4.42 | 4.42 |
|  | T2D | 6.47 | 6.53 | 6.69 | 6.60 | 6.59 |
|  | Post ACS | 1.23 | 1.23 | 1.22 | 1.23 | 1.23 |
|  | Cancer | 0.58 | 0.59 | 0.59 | 0.59 | 0.59 |
|  | Post-stroke | 0.78 | 0.78 | 0.79 | 0.78 | 0.78 |
|  | CV-events | -0.09 | -0.09 | -0.09 | -0.09 | -0.09 |

Note: OSA may occur along any health state*. ACS* acute coronary syndrome, *CAD* Canadian dollars, *CV* cardiovascular, *Incr*. incremental, *NB-32* naltrexone 32/bupropion 360, *OSA* obstructive sleep apnea, *QALY* quality-adjusted life-year, *T2D* type 2 diabetes.

Supplementary Table 16. Scenario analyses (costs are 2021 CAD).

|  | Intervention Cost* | Obesity disease cost | Total cost | Total QALY | Incr. cost | Incr. QALY | ICUR (vs. next best alternative) | ICUR vs SOC |
| --- | --- | --- | --- | --- | --- | --- | --- | --- |
| **Scenario 5, excluding weight-loss effect on cancer only** | | |  |  |  |  |  |  |
| Diet and exercise | 0 | 208 540 | 208 540 | 18.165 |  |  |  |  |
| Orlistat | 1 901 | 207 697 | 209 598 | 18.199 | 1 058 | 0.03 | 30 995 | 30 995 |
| NB-32 mg | 3 335 | 207 602 | 210 938 | 18.203 | Ext. dom. | Ext. dom. | Ext. dom. | 62 121 |
| Liraglutide 3.0 mg | 5 838 | 207 035 | 212 873 | 18.233 | Ext. dom. | Ext. dom. | Ext. dom. | 63 467 |
| Semaglutide 2.4 mg | 6 736 | 206 358 | 213 094 | 18.277 | 3 496 | 0.08 | 44 534 | 40 430 |
| **Scenario 6, excluding weight-loss effect on mortality only** | | |  |  |  |  |  |  |
| Diet and exercise | 0 | 208 388 | 208 388 | 18.17 |  |  |  |  |
| Orlistat | 1 901 | 207 415 | 209 316 | 18.21 | 929 | 0.04 | 25 028 | 25 028 |
| NB-32 mg | 3 335 | 207 259 | 210 594 | 18.21 | Ext. dom. | Ext. dom. | Ext. dom. | 52 061 |
| Liraglutide 3.0 mg | 5 838 | 206 537 | 212 375 | 18.25 | Dominated | Dominated | Dominated | 52 994 |
| Semaglutide 2.4 mg | 6 735 | 205 624 | 212 359 | 18.29 | 3 043 | 0.09 | 35 713 | 32 472 |
| **Scenario 7, excluding weight-loss effect CV disease only** | | |  |  |  |  |  |  |
| Diet and exercise | 0 | 208 457 | 208 457 | 18.18 |  |  |  |  |
| Orlistat | 1 901 | 207 549 | 209 450 | 18.22 | 992 | 0.04 | 23 799 | 23 799 |
| NB-32 mg | 3 336 | 207 425 | 210 761 | 18.22 | Ext. dom. | Ext. dom. | Ext. dom. | 46 780 |
| Liraglutide 3.0 mg | 5 839 | 206 779 | 212 618 | 18.26 | Ext. dom. | Ext. dom. | Ext. dom. | 47 476 |
| Semaglutide 2.4 mg | 6 737 | 205 993 | 212 729 | 18.32 | 3 279 | 0.10 | 31 786 | 29 488 |
| **Scenario 8, excluding weight-loss effect on knee replacement surgery only** | | | | |  |  |  |  |
| Diet and exercise | 0 | 208 473 | 208 473 | 18.18 |  |  |  |  |
| Orlistat | 1 901 | 207 581 | 209 482 | 18.22 | 1 009 | 0.04 | 23 913 | 23 913 |
| NB-32 mg | 3 336 | 207 464 | 210 800 | 18.23 | Ext. dom. | Ext. dom. | Ext. dom. | 46 442 |
| Liraglutide 3.0 mg | 5 839 | 206 838 | 212 677 | 18.27 | Ext. dom. | Ext. dom. | Ext. dom. | 47 105 |
| Semaglutide 2.4 mg | 6 737 | 206 081 | 212 818 | 18.32 | 3 336 | 0.10 | 31 878 | 29 589 |
| **Scenario 9, all subjects continue independent of response, but have a per cycle discontinuation + trial product estimand + next-line BaS** | | | | | | | | |
| Diet and exercise | 0 | 209 980 | 209 980 | 18.26 |  |  |  |  |
| Orlistat | 2 997 | 208 555 | 211 551 | 18.32 | 1 571 | 0.06 | 25 487 | 25 487 |
| Liraglutide 3.0 mg | 7 792 | 208 180 | 215 972 | 18.34 | Ext dom. | Ext dom. | Ext dom. | 71 242 |
| NB-32 mg | 6 307 | 208 073 | 214 380 | 18.35 | Ext dom. | Ext dom. | Ext dom. | 46 679 |
| Semaglutide 2.4 mg | 8 230 | 207 459 | 215 689 | 18.40 | 4 138 | 0.08 | 50 038 | 39 553 |
| **Scenario 10, all subjects continue independent of response, comparison based on the head-to-head trial STEP 8** | | | | | | | | |
| Liraglutide 3.0 mg | 7 748 | 179 302 | 187 051 | 19.425 |  |  |  |  |
| Semaglutide 2.4 mg | 8 177 | 178 098 | 186 274 | 19.507 | -776 | 0.08 | Dominant | NA |
| **Scenario 11, all subjects continue independent of response + excluding weight-loss effect on complications and mortality (combined)** | | | | | | | | |
| Diet and exercise | 0 | 208 482 | 208 482 | 18.16 |  |  |  |  |
| NB-32 mg | 6 305 | 206 760 | 213 065 | 18.20 | Ext dom. | Ext dom. | Ext dom. | 105 692 |
| Orlistat | 3 591 | 206 952 | 210 543 | 18.20 | 2 061 | 0.046 | 44 683 | 44 683 |
| Liraglutide 3.0 mg | 7 738 | 206 482 | 214 220 | 18.21 | Ext dom. | Ext dom. | Ext dom. | 104 348 |
| Semaglutide 2.4 mg | 8 165 | 205 978 | 214 143 | 18.24 | 3 600 | 0.04 | 95 142 | 67 426 |

***In the societal perspective, intervention costs are accounted for only once, as either private/public

CAD: Canadian dollars

Dominated: An intervention that is both more costly and less effective than a comparator. A dominated intervention does not offer good value for

money and is therefore excluded from the calculation of ICURs.

Ext Dom: Extendedly dominated: An intervention that is excluded because an alternative intervention can deliver greater QALY gains for a lower ICUR. *BaS* bariatric surgery, *CV* cardiovascular, *Incr.* incremental, *ICUR* incremental cost-utility ratio, *NA* not applicable*, NB*-32 naltrexone 32 mg /buprop­ion, *QALY* quality-adjusted life-year, *SOC* standard of care.

References

1. Lopes S, Meincke HH, Lamotte M, Olivieri AV, Lean MEJ. A novel decision model to predict the impact of weight management interventions: the Core Obesity Model. Obes Sci Pract. 2021;7:269–80. doi:10.1002/osp4.495

2. Novo Nordisk. Data on file: Statistical outputs - heewe017 (Fig 2). In, 2019.

3. Novo Nordisk. Data on file: STEP-2 CTR, Trial ID: NN9536-4374. In, 2020.

4. Wharton S, Liu A, Pakseresht A, Nørtoft E, Haase CL, Mancini J et al. Real-World Clinical Effectiveness of Liraglutide 3.0 mg for Weight Management in Canada. Obesity (Silver Spring). 2019;27:917-24. doi:10.1002/oby.22462

5. Ghusn W, De la Rosa A, Sacoto D, Cifuentes L, Campos A, Feris F et al. Weight Loss Outcomes Associated With Semaglutide Treatment for Patients With Overweight or Obesity. JAMA Network Open. 2022;5:e2231982-e. doi:10.1001/jamanetworkopen.2022.31982

6. U.S. Food and Drug Administration. Wegovy prescribing information. 2021. <https://www.accessdata.fda.gov/drugsatfda_docs/label/2021/215256s000lbl.pdf>. Accessed 10 Jun, 2022.

7. Rubino D, Abrahamsson N, Davies M, Hesse D, Greenway FL, Jensen C et al. Effect of continued weekly subcutaneous semaglutide vs placebo on weight loss maintenance in adults with overweight or obesity: the STEP 4 randomized clinical trial. JAMA. 2021;325:1414–25. doi:10.1001/jama.2021.3224

8. Wilding JPH, Batterham RL, Davies M, Van Gaal LF, Kandler K, Konakli K et al. Weight regain and cardiometabolic effects after withdrawal of semaglutide: The STEP 1 trial extension. Diabetes, Obesity and Metabolism. 2022;24:1553-64. doi:<https://doi.org/10.1111/dom.14725>

9. Ara R, Blake L, Gray L, Hernández M, Crowther M, Dunkley A et al. What is the clinical effectiveness and cost-effectiveness of using drugs in treating obese patients in primary care? A systematic review. Health Technol Assess. 2012;16:iii–xiv, 1–195.

10. Obesity Canada. Report card on access to obesity care: Obesity treatment for adults in Canada 2019, 2019. <https://obesitycanada.ca/research/report-card/>. URL|. Accessed Access.Date, Access.year.

11. Tarride J-E, Doumouras AG, Hong D, Paterson JM, Tibebu S, Perez R et al. Association of Roux-en-Y Gastric Bypass With Postoperative Health Care Use and Expenditures in Canada. JAMA Surgery. 2020;155:e201985-e. doi:10.1001/jamasurg.2020.1985

12. Sjöström L, Lindroos AK, Peltonen M, Torgerson J, Bouchard C, Carlsson B et al. Lifestyle, diabetes, and cardiovascular risk factors 10 years after bariatric surgery. N Engl J Med. 2004;351:2683-93. doi:10.1056/NEJMoa035622

13. Canadian Institute for Health Information (CIHI). Bariatric surgery in Canada 2014. <https://secure.cihi.ca/free_products/Bariatric_Surgery_in_Canada_EN.pdf>. URL|. Accessed Access.Date, Access.year.

14. Demssie YN, Jawaheer J, Farook S, New JP, Syed AA. Metabolic outcomes 1 year after gastric bypass surgery in obese people with type 2 diabetes. Med Princ Pract. 2012;21:125-8. doi:10.1159/000331793

15. Kral P, Holst-Hansen T, Olivieri AV, Ivanescu C, Lamotte M. Data on file: The association of body mass index (BMI) and health-related quality-of-life (HRQoL): data from two weight-loss interventional studies. In, 2022.

16. Søltoft F, Hammer M, Kragh N. The association of body mass index and health-related quality of life in the general population: data from the 2003 Health Survey of England. Qual Life Res. 2009;18:1293–9. doi:10.1007/s11136-009-9541-8

17. Holmes M. Data on file: Literature review for evidence to populate the Novo obesity model. Final report - The University Of Sheffield. In, 2017.

18. Cederholm J, Eeg-Olofsson K, Eliasson B, Zethelius B, Nilsson PM, Gudbjörnsdottir S et al. Risk prediction of cardiovascular disease in type 2 diabetes: a risk equation from the Swedish National Diabetes Register. Diabetes Care. 2008;31:2038–43.

19. Sjöström L. Review of the key results from the Swedish Obese Subjects (SOS) trial – a prospective controlled intervention study of bariatric surgery. J Intern Med. 2013;273:219–34. doi:<https://doi.org/10.1111/joim.12012>

20. NICE. Semaglutide for managing overweight and obesity [ID3850] -Public committee slides: ACM2 (slide 35). 2022. <https://www.nice.org.uk/guidance/indevelopment/gid-ta10765/documents>. Accessed 17 Jun, 2022.

21. Hippisley-Cox J, Coupland C. Development and validation of QDiabetes-2018 risk prediction algorithm to estimate future risk of type 2 diabetes: cohort study. BMJ. 2017;359:j5019.

22. Young T, Shahar E, Nieto FJ, Redline S, Newman AB, Gottlieb DJ et al. Predictors of sleep-disordered breathing in community-dwelling adults: the Sleep Heart Health Study. Arch Intern Med. 2002;162:893–900.

23. Wendelboe AM, Hegmann KT, Biggs JJ, Cox CM, Portmann AJ, Gildea JH et al. Relationships between body mass indices and surgical replacements of knee and hip joints. Am J Prev Med. 2003;25:290–5. doi:10.1016/s0749-3797(03)00218-6

24. Adams KF, Leitzmann MF, Albanes D, Kipnis V, Mouw T, Hollenbeck A et al. Body mass and colorectal cancer risk in the NIH–AARP cohort. Am J Epidemiol. 2007;166:36–45. doi:10.1093/aje/kwm049

25. Renehan AG, Tyson M, Egger M, Heller RF, Zwahlen M. Body-mass index and incidence of cancer: a systematic review and meta-analysis of prospective observational studies. Lancet. 2008;371:569–78. doi:10.1016/s0140-6736(08)60269-x

26. Renehan AG, Zwahlen M, Egger M. Adiposity and cancer risk: new mechanistic insights from epidemiology. Nat Rev Cancer. 2015;15:484–98. doi:10.1038/nrc3967

27. Lopes S, Johansen P, Lamotte M, McEwan P, Olivieri AV, Foos V. External validation of the core obesity model to assess the cost-effectiveness of weight management interventions. Pharmacoeconomics. 2020;38:1123–33. doi:10.1007/s40273-020-00941-3

28. Statistics Canada. Life expectancy and other elements of the complete life table, three-year estimates, Canada, all provinces except Prince Edward Island. Table 13-10-0114-01. 2022. <https://www150.statcan.gc.ca/t1/tbl1/en/tv.action?pid=1310011401>. Accessed 18 Feb, 2022.

29. British Heart Foundation Health Promotion Research Group. Coronary heart disease statistics: a compendium of health statistics. 2012 edition. 2012. <https://www.bhf.org.uk/~/media/files/publications/research/2012_chd_statistics_compendium.pdf>. Accessed 10 Jun, 2022.

30. Johansson S, Rosengren A, Young K, Jennings E. Mortality and morbidity trends after the first year in survivors of acute myocardial infarction: a systematic review. BMC Cardiovasc Disord. 2017;17:53.

31. Brammås A, Jakobsson S, Ulvenstam A, Mooe T. Mortality after ischemic stroke in patients with acute myocardial infarction: predictors and trends over time in Sweden. Stroke. 2013;44:3050–5.

32. Cancer Research UK. Statistics by cancer type. <https://www.cancerresearchuk.org/health-professional/cancer-statistics/statistics-by-cancer-type>. Accessed 22 Jul, 2022.

33. Novo Nordisk Canada Inc. SAXENDA® product monograph. 2021. <https://www.novonordisk.ca/content/dam/nncorp/ca/en/products/Saxenda%20PM%20English%20-%20Marketed%20-%2025%20February%202021.pdf>. Accessed 10 Apr, 2022.

34. IQVIA. Delta PA (proprietary of IQVIA; requires access rights). 2022. <https://www.customerportal.iqvia.com/sites/portal/products>. Accessed 25 Feb, 2022.

35. Cheplapharm Arzneimittel GmbH. XENICAL® product monograph. 2017. <https://pdf.hres.ca/dpd_pm/00041463.PDF>. Accessed 22 Jul, 2022.

36. Bausch Health Canada Inc. CONTRAVE® product monograph. 2020. <https://pdf.hres.ca/dpd_pm/00056659.PDF>. Accessed 23 Mar, 2022.

37. IQVIA. Delta PA – Canadian formulary and wholesale drug prices (accessible with subscription). 2022. <https://www.iqvia.com/>. Accessed 12 Jul, 2022.

38. Wharton S, Lau DCW, Vallis M, Sharma AM, Biertho L, Campbell-Scherer D et al. Obesity in adults: a clinical practice guideline. CMAJ. 2020;192:E875–91. doi:10.1503/cmaj.191707

39. Ontario Ministry of Health and Long-Term Care. Physician Services – schedule of benefits. Physician Services Under the Health Insurance Act. 2022. <https://www.health.gov.on.ca/en/pro/programs/ohip/sob/>. Accessed 23 Jun, 2022.

40. Ontario Ministry of Health and Long-Term Care. Laboratory services. Schedule of benefits for laboratory services. 2020. <https://www.health.gov.on.ca/en/pro/programs/ohip/sob/>. Accessed 23 Jun, 2022.

41. Weight Watchers. Pricing: digital membership. 2022. <https://www.weightwatchers.com/ca/en/plans>. Accessed 22 Jun, 2022.

42. Hôtel-Dieu Grace Healthcare. Medical weight management. 2018. <https://www.hdgh.org/medicalweightmanagement>. Accessed 18 Aug, 2021.

43. Freedieting. Nutrisystem diet. 2021. <https://www.freedieting.com/nutrisystem>. Accessed 18 Aug, 2021.

44. Jenny Craig. How much does Jenny Craig cost? 2019. <https://community.jennycraig.com/healthy-habits-blog/eat-well/how-much-does-jenny-craig-cost/>. Accessed 18 Aug, 2021.

45. Ontario Ministry of Health and Long-Term Care. Ambulance services billing. 2012. <https://www.health.gov.on.ca/en/public/publications/ohip/amb.aspx>. Accessed 18 Aug, 2021.

46. CADTH. Common Drug Review - Pasireotide (Signifor), 2015. <https://www.cadth.ca/sites/default/files/cdr/clinical/SR0372_Signifor_CL_Report.pdf>. URL|. Accessed Access.Date, Access.year.

47. CADTH. Optimal use reports Ottawa (ON), 2011. <https://www.ncbi.nlm.nih.gov/books/NBK168999/>. URL|. Accessed Access.Date, Access.year.

48. Aronson R, Galstyan G, Goldfracht M, Al Sifri S, Elliott L, Khunti K. Direct and indirect health economic impact of hypoglycaemia in a global population of patients with insulin-treated diabetes. Diabetes Res Clin Pract. 2018;138:35-43. doi:10.1016/j.diabres.2018.01.007

49. CADTH. Therapeutic review: New drugs for type 2 diabetes: Second-line therapy – Science report, 2017. <https://www.cadth.ca/sites/default/files/pdf/TR0012_T2D_Science_Report.pdf>. URL|. Accessed Access.Date, Access.year.

50. Canadian Diabetes Association. The burden of out-of-pocket costs for Canadians with diabete. 2015. <https://www.yumpu.com/en/document/read/42983786/burden-of-out-of-pocket-costs-for-canadians-with-diabetes>. Accessed 3 Oct, 2022.

51. Alberta RWE Consortium. Data on file: Comparative cost analysis of adult obesity in Albertaovo. In, 2021.

52. Longo CJ, Fitch MI, Loree JM, Carlson LE, Turner D, Cheung WY et al. Patient and family financial burden associated with cancer treatment in Canada: a national study. Support Care Cancer. 2021;29:3377–86. doi:10.1007/s00520-020-05907-x

53. de Oliveira C, Pataky R, Bremner KE, Rangrej J, Chan KK, Cheung WY et al. Phase-specific and lifetime costs of cancer care in Ontario, Canada. BMC Cancer. 2016;16:809. doi:10.1186/s12885-016-2835-7

54. Iragorri N, de Oliveira C, Fitzgerald N, Essue B. The out-of-pocket cost burden of cancer care-a systematic literature review. Curr Oncol. 2021;28:1216–48.

55. Dhalla IA, Smith MA, Choudhry NK, Denburg AE. Costs and benefits of free medications after myocardial infarction. Healthc Policy. 2009;5:68–86.

56. Cohen DM, D.J. Tugwell, P. Sanmartind, C. Ramsaye, T. Direct healthcare costs of acute myocardial infarction in Canada’s elderly across the continuum of care. The Journal of the Economics of Ageing. 2014;3:44-9.

57. Wagner M, Lindgren P, Merikle E, Goetghebeur M, Jönsson B. Economic evaluation of high-dose (80 mg/day) atorvastatin treatment compared with standard-dose (20 mg/day to 40 mg/day) simvastatin treatment in Canada based on the Incremental Decrease in End-Points Through Aggressive Lipid-Lowering (IDEAL) trial. Can J Cardiol. 2009;25:e362–9. doi:10.1016/s0828-282x(09)70159-x

58. McGillion MH, Croxford R, Watt-Watson J, Lefort S, Stevens B, Coyte P. Cost of illness for chronic stable angina patients enrolled in a self-management education trial. Can J Cardiol. 2008;24:759–64. doi:10.1016/s0828-282x(08)70680-9

59. Canada's Drug and Health Technology Agency. New drugs for type 2 diabetes: second-line therapy – science report. In: CADTH Therapeutic Review, Volume 4, Issue No. 1b, 2017.

60. Goeree R, Blackhouse G, Petrovic R, Salama S. Cost of stroke in Canada: a 1-year prospective study. J Med Econ. 2005;8:147–67. doi:10.3111/200508147167

61. Yu AYX, Krahn M, Austin PC, Rashid M, Fang J, Porter J et al. Sex differences in direct healthcare costs following stroke: a population-based cohort study. BMC Health Serv Res. 2021;21:619. doi:10.1186/s12913-021-06669-w

62. Wein T, Mancini J, Rogoza RM, Pericleous L. New view on the Canadian burden of stroke: productivity loss in adults who return to work. Can J Neurol Sci. 2021;48:421–4. doi:10.1017/cjn.2020.192

63. Tawfik A, Wodchis WP, Pechlivanoglou P, Hoch J, Husereau D, Krahn M. Using phase-based costing of real-world data to inform decision-analytic models for atrial fibrillation. Appl Health Econ Health Policy. 2016;14:313–22. doi:10.1007/s40258-016-0229-2

64. Mittmann N, Seung SJ, Hill MD, Phillips SJ, Hachinski V, Coté R et al. Impact of disability status on ischemic stroke costs in canada in the first year. Can J Neurol Sci. 2012;39:793–800. doi:10.1017/S0317167100015638

65. Kim J, Tran K, Seal K, Almeida F, Ross G, Messier MR et al. Interventions for the treatment of obstructive sleep apnea in adults: a health technology assessment. In: CADTH Optimal Use Report, Volume 6, Issue 1b, 2017.

66. Pendharkar SR, Povitz M, Bansback N, George CFP, Morrison D, Ayas NT et al. Testing and treatment for obstructive sleep apnea in Canada: funding models must change. CMAJ. 2017;189:E1524–8. doi:10.1503/cmaj.170393

67. Tan MC, Ayas NT, Mulgrew A, Cortes L, FitzGerald JM, Fleetham JA et al. Cost-effectiveness of continuous positive airway pressure therapy in patients with obstructive sleep apnea-hypopnea in British Columbia. Can Respir J. 2008;15:159–65. doi:10.1155/2008/719231

68. Ontario Ministry of Health and Long-Term Care Health Data Branch. Ontario Case Costing Initiative - data catalog (requires access rights). 2017. <https://hsim.health.gov.on.ca/hdbportal/> Accessed 14 Feb, 2022.

69. Tarride JE, Doumouras AG, Hong D, Paterson JM, Tibebu S, Perez R et al. Association of Roux-en-Y gastric bypass with postoperative health care use and expenditures in Canada. JAMA Surg. 2020;155:e201985. doi:10.1001/jamasurg.2020.1985

70. Sheppard CE, Lester EL, Chuck AW, Birch DW, Karmali S, de Gara CJ. The economic impact of weight regain. Gastroenterol Res Pract. 2013;2013:379564. doi:10.1155/2013/379564

71. Lester ELW, Padwal RS, Birch DW, Sharma AM, So H, Ye F et al. The real-world cost-effectiveness of bariatric surgery for the treatment of severe obesity: a cost-utility analysis. CMAJ Open. 2021;9:E673–9. doi:10.9778/cmajo.20200188

72. Canadian Institute for Health Information. Bariatric surgery in Canada. 2014. <https://secure.cihi.ca/free_products/Bariatric_Surgery_in_Canada_EN.pdf>. Accessed 14 Jul, 2021.

73. Marshall DA, Wasylak T, Khong H, Parker RD, Faris PD, Frank C. Measuring the value of total hip and knee arthroplasty: considering costs over the continuum of care. Clin Orthop Relat Res. 2012;470:1065–72. doi:10.1007/s11999-011-2026-4

74. Canadian Institute for Health Information. Hip and knee replacements in Canada: CJRR Annual Report. 2020–2021. 2022. <https://www.cihi.ca/sites/default/files/document/hip-knee-replacements-in-canada-cjrr-annual-report-2020-2021-en.pdf>. Accessed 14 Jul, 2022.

75. Gough SC, Kragh N, Ploug UJ, Hammer M. Impact of obesity and type 2 diabetes on health-related quality of life in the general population in England. Diabetes Metab Syndr Obes. 2009;2:179–84. doi:10.2147/dmsott.s7088

76. Sullivan PW, Slejko JF, Sculpher MJ, Ghushchyan V. Catalogue of EQ-5D scores for the United Kingdom. Med Decis Making. 2011;31:800–4. doi:10.1177/0272989x11401031

77. Campbell J, McGarry LA, Shikora SA, Hale BC, Lee JT, Weinstein MC. Cost-effectiveness of laparoscopic gastric banding and bypass for morbid obesity. Am J Manag Care. 2010;16:e174–87.

78. Clarke P, Gray A, Holman R. Estimating utility values for health states of type 2 diabetic patients using the EQ-5D (UKPDS 62). Med Decis Making. 2002;22:340–9. doi:10.1177/0272989x0202200412

79. National Institute for Health and Care Excellence. Naltrexone–bupropion for managing overweight and obesity. Technology appraisal guidance [TA494]. 2017. <https://www.nice.org.uk/guidance/ta494>. Accessed 22 Jul, 2022.

80. Foos V, McEwan, P. Conversion of hypoglycemia utility decrements from categorical units reflecting event history into event specific disutility scores applicable to diabetes decision models. Value Health. 2018;21:S223.
